# Preliminary Identification of PFAS and Other Emerging Contaminants in the French Broad River, NC Post-Hurricane Helene

**DOI:** 10.3390/toxics13110905

**Published:** 2025-10-22

**Authors:** Imari Walker-Franklin, Samantha Blake, Evan Thorp, Shea Tuberty

**Affiliations:** 1 RTI International, Durham, NC 27713, USA; 2 Department of Biology, Appalachian State University, Boone, NC 28608, USA

**Keywords:** environmental monitoring, extreme weather events, non-targeted analysis (NTA), per- and polyfluoroalkyl substances (PFAS), post-disaster assessment, water quality

## Abstract

Hurricanes are increasingly impacting inland water systems, yet their role in mobilizing anthropogenic contaminants remains insufficiently characterized. This study presents a preliminary assessment of organic contaminant loading in the French Broad River, North Carolina, 15 days after flooding from Hurricane Helene. Surface water samples from five sites were analyzed using liquid chromatography–high-resolution mass spectrometry. Targeted analysis quantified 11 per- and polyfluoroalkyl substances (PFASs), with summed concentrations ranging from 1.49 to 70.8 ng/L. One downstream site exhibited the highest burden, where PFOSs and PFOA exceeded U.S. EPA drinking water maximum contaminant levels. Non-targeted analysis (NTA) and suspect screening identified 468 compounds, with 96 structurally annotated at high confidence. Of these, a large proportion were associated with medium to high hazard potential, particularly for acute aquatic toxicity (69%), developmental toxicity (64%), mutagenic genotoxicity (49%), endocrine disruption (35%), skin irritation (27%), eye irritation (26%), and carcinogenicity (17%). Four EPA priority pollutants—4-nitrophenol, 2,4,6-trichlorophenol, pentachlorophenol, and dibutyl phthalate—were also detected. Site-specific chemical profiles suggested inputs from flood-damaged wastewater infrastructure and plastic debris. These findings highlight the susceptibility of freshwater systems to contaminant mobilization during extreme flooding and demonstrate the value of combining NTA and cheminformatics for post-disaster monitoring.

## 1. Introduction

The French Broad River (FBR) basin is one of the largest river systems in North Carolina (NC), with over 350 km (km) of river flowing through Western NC (WNC) and into Tennessee (TN). The FBR runs in close proximity to areas used for tourism, agriculture, manufacturing, water sports, and fishing, making it a vital resource for both ecological and economic activities [1]. On 27 September 2024, Hurricane Helene struck Western North Carolina (WNC) and became the US region most severely impacted by the storm [2,3,4,5]. The combination of heavy rainfall and high winds triggered flooding, treefalls, landslides, and tornadoes, resulting in mass destruction of water and sewer systems, homes and businesses [6,7].

During Hurricane Helene, the FBR basin crested at approximately 7.3 meters (m), setting an all-time record and surpassing the historic flood levels of 1916, 1940, and 2004 [8,9]. This unprecedented rise in floodwaters is proposed to have inundated the FBR with a wide range of anthropogenic contaminants, including chemicals associated with human sewage, roadways, buildings, industrial materials, agricultural runoff, and other waste sources [10]. Given the variety of both new and legacy materials likely to enter the FBR through runoff from the storm, the primary contaminants of concern include pathogens, heavy metals, per- and polyfluoroalkyl substances (PFAS), polymer-associated chemicals, pesticides, pharmaceuticals and personal care products [11,12,13]. The FBR’s significant ecological value, extensive human use, and history of severe flood events make it a priority site for monitoring contaminant mobilization following natural disasters.

Most post-hurricane environmental monitoring of surface waters has relied on targeted analytical methods to quantify a predefined list of contaminants [14,15,16]. However, targeted analysis is inherently limited and may miss the hundreds to thousands of emerging and uncharacterized chemicals introduced during flooding events. More recently, research studies have begun incorporating suspect screening and non-targeted analysis (NTA) using high-resolution mass spectrometry (HRMS) to broaden contaminant detection in post-disaster water samples [17,18]. Suspect screening involves querying data against curated databases of potentially hazardous compounds. NTA enables the prioritization of chemical features that may be more abundant or significant than those included in targeted or suspect lists [19,20,21]. NTA relies on accurate mass and spectral data to structurally annotate and identify novel compounds, offering a more comprehensive view of chemical contamination following extreme weather events [22]. However, very few studies have provided chemical hazard context for contaminants detected post-hurricane destruction [23,24].

The primary objective of this study was to inform disaster-response monitoring efforts by applying a hazard profiling framework that integrates both targeted analysis and NTA with cheminformatics-based hazard screening. We first employed a combined analytical approach including targeted quantitation of PFAS, suspect screening, and NTA to conduct a preliminary evaluation of both regulated and emerging environmental contaminants in water samples collected from the FBR basin 15 days after Hurricane Helene. Subsequently, these polar organic micropollutants were further prioritized using hazard data from the EPA cheminformatics module. We hypothesized that the potentially hazardous contaminant loading would be greatest in areas with observable increased levels of debris and infrastructure damage.

## 2. Materials and Methods

### 2.1. Site Locations and Sample Collection

**Figure 1:**
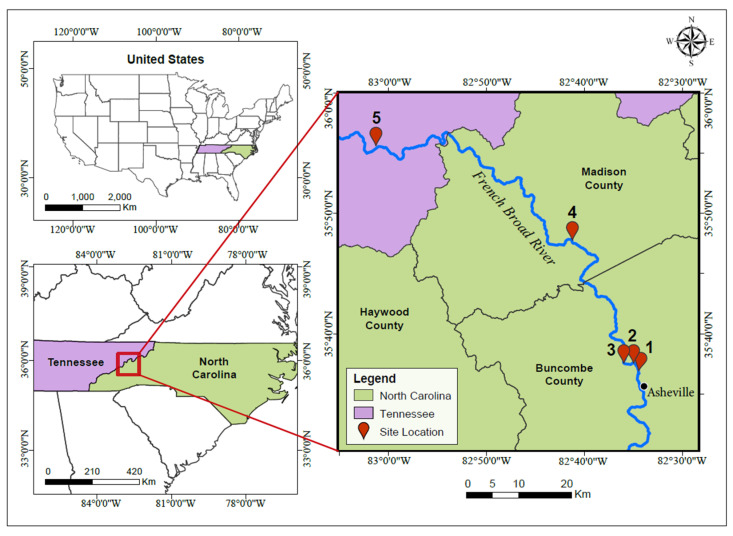
Location of sampling sites along the French Broad River in North Carolina (NC) and Tennessee (TN). Samples were taken along the river flowing north from Asheville, NC into Knoxville, TN. These five locations numbered 1–5 were accessible sites for sampling and near known areas for tourism and recreation year-round.

All samples were collected on 12 October 2024, 15 days post-Hurricane Helene, the soonest accessible time point to access these locations safely. Surface water samples were collected from the French Broad River flowing north at five locations along an 80-km transect. The sites are displayed in the map in Figure 1 with GPS coordinates as follows: Site 1 = 35.614123, −822.576467; Site 2 = 35.625429, −82.583083; Site 3 = 35.626082, −82.601331; Site 4A = 35.795471, −82.685632; Site 4B = 35.796641, −82.687854; Site 5 = 35.895328, −82.823953. The locations were selected based on accessibility constraints due to storm-damaged roads and bridges. These locations were also strategically positioned downstream of towns, businesses, and popular recreational access points (e.g., fishing, swimming, kayaking, inner tubing). Sample Site 4 was divided into two sub-sites, 4A and 4B, to include an additional sample collected near a visibly damaged wastewater treatment plant. Site 4B represents an area of observed wastewater overflow entering the river. Accordingly, downstream sample Site 5 was proposed to represent a mixed input of flood- and infrastructure-related contamination from the river and other creek and branch contributions. One field blank of deionized water was utilized to assess potential contamination during sampling and handling.

Due to sampling constraints, the sampling study design was intended to reflect an exploratory sampling strategy meant to inform longer-term and more standardized monitoring thus only single replicates were obtained. All samples were immediately stored on ice in a dark cooler, transported to a laboratory, and then stored in a 4 °C refrigerator until extraction and analysis on 8 November 2024. Additional sampling information and water quality parameters of the FBR during the time frame of sample collection are provided in the Supplementary Information (SI, Tables S1–S3).

### 2.2. Materials

All reagents used for water extraction and mobile phase preparation were of 99% purity or higher. Ammonium acetate was purchased from JT Baker (Radnor, PA, USA). Ammonium hydroxide was purchased from ACROS Organics (Waltham, MA, USA). High-purity grade solvents methanol, acetonitrile, and water were used for extraction and mobile phases. HPLC grade water and acetonitrile was purchased from Thermo Fisher (Waltham, MA, USA) and HPLC grade methanol was purchased from Birch biotech (Portland, OR, USA). Ultrapure water (18.2 MΩ) was used for field blanks and rinsing and cleaning glassware (Hydro Picopure UV plus, Durham, NC, USA). All individual compounds and chemical mixtures used for internal standards, identification, quantification or for quality control had a purity greater than 95% and are detailed in SI Table S4.

### 2.3. Sample Preparation

Water samples (*n* = 5), field blank (*n* = 1), laboratory reagent blank (*n* = 1), and laboratory PFAS fortified blank at 5 ng/mL (*n* = 1) were processed via solid phase extraction (SPE). SPE protocols followed EPA Method 533 to prioritize accurate quantitation of anionic PFAS [25]. Samples and blanks were individually filtered through a 45 mm 0.7 µM pore Whatman Glass Fiber Filter (Sigma Aldrich, Burlington, MA, USA) while maintaining the pH between 6–8 with ammonium acetate. Following filtration, samples and blanks were spiked with isotopically labeled PFAS standards. These samples were then loaded at 5 mL/min, onto an automated SPE system (Promochrom, Richmond, BC, Canada), equipped with a preconditioned Phenomenex (Torrance, CA, USA) polymeric SPE cartridge [Stra-X-AW, 33 µm, 500 mg]. Samples were rinsed with 10 mL of 1 g/L ammonium acetate in water and 1 mL methanol then dried under vacuum. Extracts were eluted using 10 mL 2% ammonium hydroxide in methanol. Eluent was dried under nitrogen in a heated water bath (55–60 °C) and reconstituted with 0.5 mL 80:20 methanol:water with isotope PFAS performance standards (IPS) included. The samples were then transferred to a 1 mL Thermo Fisher SureSTART polypropylene autosampler vial (Waltham, MA, USA) and stored at −20 °C before analysis. Samples were pooled and the pools were injected in triplicate to aid in feature detection and peak area normalization. Normalization can be performed either to internal standards or to pooled QC features. We choose the pooled QC to better represent changes to signal intensities with each feature detected throughout the instrument run. This practice has been encouraged within the exposomics community (e.g., BP4NTA) as a quality control measure [26,27].

### 2.4. Sample Analysis

Sample extracts were quantified for the 25 PFAS listed in EPA Method 533 using a SCIEX ExionLC Liquid Chromatograph coupled to a Sciex 7500 triple quadrupole Mass Spectrometer (LC-MS/MS) (Framingham, MA, USA). Additionally, sample extracts were injected in a randomized sequence on a Thermo Horizon UPLC coupled to a Thermo Exploris 480 Orbitrap (UPLC-HRMS) (Waltham, MA, USA) to collect both high-resolution MS and data-dependent MS/MS data. Water extracts from the FBR were analyzed using LC-HRMS in both positive and negative electrospray ionization modes. Samples were analyzed in both polarities to detect polar organic ionizable chemical features, represented as organic compounds, present in the samples. Instrument calibration on the HRMS system was completed before the run. System suitability was verified by injecting Waters LC Suitability Standards (Milford, MA, USA) in triplicate at both the start and conclusion of the analytical run on the HRMS system. The performance of reference compounds was assessed to confirm compliance with predefined criteria for reliable data analysis. Evaluation metrics included retention time stability (variation within ±0.1 min), peak area reproducibility (relative standard deviation < 30%) and mass accuracy (within ±5 ppm). Additional instrument methods, quality assurance and quality control parameters for both mass spectrometer systems are described in the SI Tables S5–S8.

NTA workflow processing was completed using Thermo Compound Discoverer v. 3.3 (Waltham, MA, USA) for feature alignment, peak area integration, and chemical formula assignment. A feature is a data point representing a chemical entity defined by retention time (RT), mass-to-charge ratio (*m*/*z*) and its peak area abundance when detected on the LC-HRMS system. Feature peak area abundance that was five-fold higher in the sample than the solvent, reagent, and field blanks were retained for annotation. Peak areas detected for each feature were normalized using the pooled samples as quality control samples.

Feature annotation with known chemical structures or molecular formulas followed the Schymanski framework for confidence level assignment [17]. Level 1 confidence matches utilized chemical reference standards that had corresponding retention time, precursor ion *m*/*z* mass accuracy (<5 ppm) and matching MS/MS fragmentation to the feature. Level 2 confidence in annotation was assigned to features with precursor ion *m*/*z* mass accuracy (<5 ppm) and MS/MS fragmentation spectral library matches greater than 75% to the MzCloud database (Highchem, Bratislava, Slovakia) or in house spectral libraries [28]. Level 3 annotations were for in silico MS/MS fragmentation spectral matches using Thermo Fisher Mass Frontier and attaining a Fish score greater than 50 or a PFAS class coverage score greater than 6. Features were assigned Level 4 annotation if they only had assigned molecular formulas, or predicted formula matches or exact mass matches (<5 ppm) to a mass list or selected database [29]. Level 5 annotations only had accurate mass details and no other annotation for formula and name. Additional details on the databases, spectral libraries and mass lists used for the data processing workflow are provided in the SI Table S9.

### 2.5. Hazard Classifications of Annotations

The EPA Cheminformatics module was created as a tool for comparing hazardous of compounds by inputting chemicals identifiers (DTXSID) to generate a hazard profile across 20 human health and ecological endpoints (version: DEV, build: 2023-03-09) [30]. This database compiled and integrated hazard data from authoritative regulatory hazard lists (i.e., EU ECHA, DHH NTP Carcinogens report, CA Prop 65 list), screening sources (i.e., Health Canada Priority Substances Lists, US EPA Toxic Substances Control Act (TSCA), and predictive Quantitative Structure–Activity Relationship (QSAR) models (i.e., EPA toxicity estimation software tool version 5.1.2). This module provides hazard classifications ranging from none, inconclusive, low, medium, and high, to very high [31]. Each classification is accompanied by annotations that cite the authority of the determination, including toxicology data from government health agencies as well as experimental or predicted toxicology values. During the publication, the hazard database had 990,000 score records created for 85,000 chemicals [30].

## 3. Results and Discussion

### 3.1. Targeted PFAS Analysis

**Figure 2:**
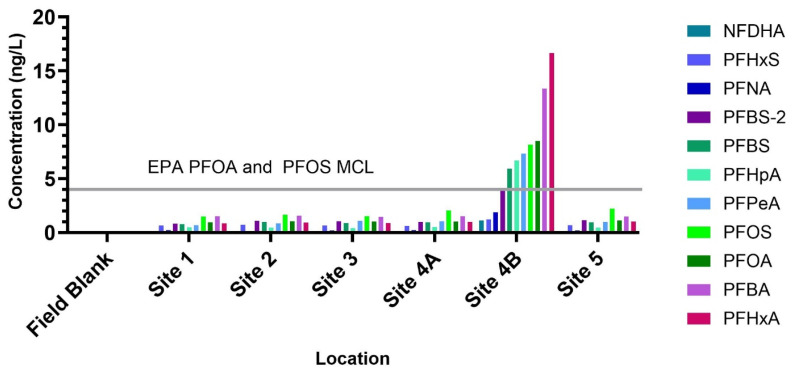
Ten PFAS concentrations (ng/L) are plotted along each site sampled along with the EPA PFOA and PFOS maximum contaminant limit (MCL) at 4 ng/L. PFBS-2 is a coeluting peak with PFBS, which is quantified with a different confirmation ion. See SI Figure S4 for more details.

Targeted analysis of 25 PFAS compounds detected 11 individual PFAS across all sample locations (Figure 2). Site 4B exhibited the highest overall PFAS burden, with a summed PFAS concentration of 70.8 ng/L—an order of magnitude greater than the other sites (Site 1: 1.49 ng/L; Site 2: 3.66 ng/L; Site 3: 4.52 ng/L; Site 4A: 5.08 ng/L; Site 5: 5.31 ng/L). At Site 4B, PFOA (8.48 ng/L) and PFOS (8.15 ng/L) both exceeded the EPA maximum contaminant level (MCL) for drinking water (4 ng/L). Additional PFAS detected at this site included PFHxA (16.6 ng/L), PFBA (13.3 ng/L), PFPeA (7.33 ng/L), PFHpA (6.70 ng/L), PFBS (5.92 ng/L), PFNA (1.6 ng/L), PFHxS (1.21 ng/L), and NFDHA (1.10 ng/L). PFAS are widely used in consumer and industrial products, including pesticides, cosmetics, food packaging, dental floss, non-stick cookware, cleaning agents, firefighting foams, and textiles [32,33]. Such products may have been mobilized into the river along with storm debris, consistent with previous observations of elevated PFOS concentrations in Florida surface waters during Hurricane Dorian (2019) and increased PFOA concentrations in Puerto Rico surface waters following Hurricane Maria (2017) [14,34]. Importantly, these concentrations are relevant given the well-documented hazards of PFAS, including their persistence, bioaccumulation, toxicity to humans and wildlife, and potential links to cancer, endocrine disruption, and immune system effects [13,35].

PFOS concentrations exceeded the EPA lifetime health advisory level for drinking water (0.02 ng/L) at all sites. Moreover, PFOS was the most abundant PFAS at all sites but 4B, with concentrations ranging from 1.49 to 2.22 ng/L. While PFAS has been recognized as a common anthropogenic contaminant in North Carolina Watersheds, recent work by NCDEQ (2022) quantified 28 PFASs in French Broad River basin reservoirs and reported no detectable PFASs in Allen Creek, Beetree, or Burnett reservoirs [36]. In contrast, the neighboring Yadkin–Pee Dee River system contained 10 detectable PFASs in 2020, with the highest concentrations observed for PFOS (4.34 ng/L), PFHxA (4.37 ng/L), PFHpA (5.79 ng/L), and PFOA (8.07 ng/L) [37].

While no federal regulatory standards currently exist for PFASs in surface waters, it is notable that Site 1 is located approximately 32 km downstream from a drinking water intake for the Asheville system. Prior to the storm, testing of Asheville’s treated drinking water reported no detectable PFASs. More recently, the City of Asheville sampled the French Broad River intake both before Hurricane Helene (4 September 2024) and after the flood event (9 December 2024), finding no detectable levels of PFOA, PFOS, PFHxS, PFNA, PFBS, or HFPO-DA [38]. The differences between our results and these municipal surveys may reflect variations in sampling design, analytical methods, or instrument detection limits and warrant further investigation. Collectively, these findings highlight the importance of continued PFAS monitoring to ensure protection of public health.

NTA identified 61 features containing fluorine within their predicted molecular formulas, with mass accuracy within 2 ppm. Many of these features were most abundant at Site 4B. A Kendrick Mass Defect plot, aligned to CF2 units (SI Figure S1, SI Excel Data File), was used to visualize molecular trends. Of the 61 features, 34 were prioritized due to the availability of MS2 spectra. Level 3 compound annotations were assigned using features with MS2 fragments matching >800 PFAS fragments within the FluoroMatch Suite or PFAS Fine signature libraries [39]. Several annotated compounds with entries in the EPA CompTox database were predicted to have potential functional uses, including antimicrobials (e.g., 5-Chloro-4-methoxy-2-(trifluoromethyl)-1H-benzimidazole; 3-Bromo-4-[(3-fluorobenzyl)oxy]-5-methoxybenzaldehyde; xenalipin; 3-(Trifluoromethyl)phenylacetic acid; methyl difluoro(phenoxysulfonyl)acetate), emulsion stabilizers (e.g., 1,1,1,2,2,3,3,4,4,5,5,6,6,7,7-pentadecafluorotridecane), and foamers (e.g., 1,1,1,2,2,3,3,4,4,5,6,6,6-tridecafluorohexane) [40]. While many features detected here may represent novel PFAS in the FBR, annotation confidence remains low due to limited MS2 data and the absence of reference standards. This highlights a significant knowledge gap in characterizing the diversity and potential sources of PFAS in impacted watersheds.

### 3.2. Overview of Detected Chemical Features

**Figure 3:**
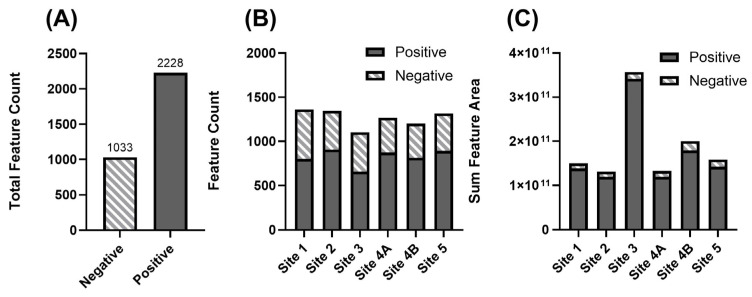
Summary of detected chemical features across sampling sites displayed as: (**A**) Total number of unique features identified across all sites, found by both positive and negative ionization modes, (**B**) Number of features detected at each individual site (positive and negative ionization modes), and (**C**) Relative chemical abundance represented by the summed integrated feature peak areas (arbitrary units) for each site, measured in both positive and negative ionization modes.

Non-targeted analysis identified 2228 features in positive ion mode and 1033 unique features in negative ion mode (Figure 3A). These feature counts were comparable to the Hurricane Florence NTA of surface waters (Feature count ranged 867–1493), [18]. The higher number of features observed in positive ionization is expected. Many environmental contaminants like pharmaceuticals readily ionize through protonation, leading to greater detection sensitivity in this mode [41]. Feature counts varied slightly across sites (Figure 3B), with Site 1 exhibiting the highest number of detected features (Site 1—1360 features; Site 2—1346 features; Site 3—1103 features; Site 4A—1267 features; Site 4B—1201 features; Site 5—1314 features).

Total numbers of detected features were compared to make preliminary assessments of total chemical loadings at each sample location. Site 3 had the lowest number of unique features detected. However, Site 3 exhibited the highest total peak area, suggesting a disproportionately high abundance of a smaller subset of compounds (Figure 3C). This indicates that Site 3 may be impacted by a limited group of dominant contaminants contributing significantly to the overall chemical burden at that location. The five most abundant positive ion features at Site 3 were high-molecular-weight compounds (ranging from 398.18 to 904.67 Da). These Level 4 annotated features had predicted molecular formulas composed of common biological elements (Chemicals: C_47_H_92_N_4_O_12_, C_19_H_30_N_4_O_8_, C_21_H_26_N_4_O_2_S, C_43_H_86_NO_7_PS, and C_36_H_66_N_6_O_6_). The high-mass, non-halogenated nature (determined by isotopic pattern and molecular formula prediction) of these features suggests a potential origin as natural products. However, the compounds were annotated as unknowns as they additionally did not match to any of the spectral libraries used in the data processing.

This analytical approach combined non-targeted feature detection with comparative peak area abundance data. It facilitated site-wise comparisons of chemical diversity and contaminant loading. Additionally, this method supports the prioritization of post storm monitoring efforts at locations. For example, the chemical abundances of various unknowns at Site 3 suggest that conventional contaminant lists may not capture the full extent of organic contaminants input into freshwater systems.

### 3.3. High-Risk Compounds Annotated via Suspect Screening and Hazard Screening

The “Result Filter” feature in Compound Discoverer 3.3 was utilized to apply specific thresholds to the dataset. Applying this feature aimed to reduce false positives and eliminate less relevant features. The filter applied customized criteria to prioritize chemical features with high-confidence spectral library matches. Additionally, the filter prioritized retaining features with exact mass (<5 ppm) or predicted molecular formula matches to established databases and mass lists, including those for pesticides, polymer additives, PFAS, and EPA-designated contaminants of emerging concern (SI Figure S2). This filtering process prioritized approximately 20% of the initially detected features (372 features in positive mode and 272 in negative mode) for subsequent hazard evaluation. Once each feature was annotated with a unique compound name and assigned a confidence level (Levels 1–4), each feature was referred to as a chemical compound.

Compounds assigned Level 1–4 confidence and linked to CAS registry numbers (267 in positive mode and 201 in negative mode) were screened using the EPA’s cheminformatics hazard module [42]. This process identified 468 compounds with available hazard information with 96 compounds structurally annotated with Level 1 or Level 2 confidence, as detailed in the SI Table S11 and SI Excel Data File. From these high level confidence annotations with available hazard data, 69% were classified as having medium to very high hazard potential for acute aquatic toxicity and 41% for chronic aquatic toxicity. For human health hazards, at least 15% of the Level 1 or Level 2 chemical annotations were classified as medium to very high hazard potential included compounds with acute mammalian oral toxicity (65%), developmental toxicity (64%), mutagenic genotoxicity (49%), endocrine disruption (35%), reproductive toxicity (28%), repeat exposure systemic toxicity (27%), skin irritation (27%), eye irritation (26%), and known or suspected carcinogenic properties (17%), and skin sensitization (16%). The chemical classes identified included polymer-associated chemicals, PFAS, pesticides, personal care products, pharmaceuticals, and wastewater tracers.

**Figure 4:**
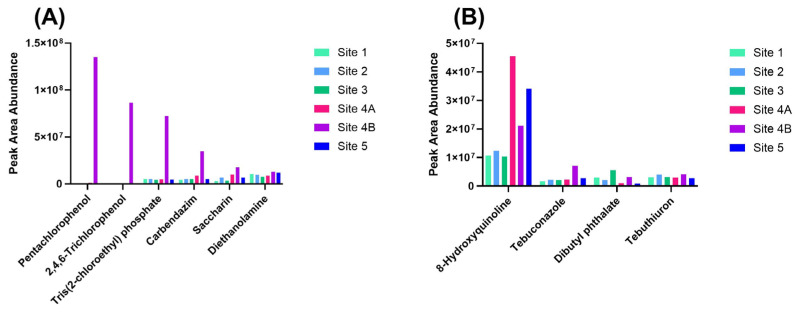
Peak area abundance (arbitrary units) of Level 1 or Level 2 chemicals listed as very high hazards from EPA cheminformatic classified authoritative data for (**A**) carcinogenicity and (**B**) acute aquatic toxicity. Chemical names are denoted by the common name listed in the EPA Cheminformatics Module. IUPAC chemical names from left to right in Figure 3A are 2,3,4,5,6-pentachlorophenol; 2,4,6-trichlorophenol; tris(2-chloroethyl) phosphate; methyl N-(1H-benzimidazol-2-yl)carbamate; 1,1-dioxo-1,2-benzothiazol-3-one; and 2-(2-hydroxyethylamino)ethanol. IUPAC chemical names from left to right for Figure 3B are: Quinolin-8-ol; 1-(4-chlorophenyl)-4,4-dimethyl-3-(1,2,4-triazol-1-ylmethyl)pentan-3-ol; Dibutyl benzene-12-dicarboxylate; and 1-(5-tert-butyl-1,3,4-thiadiazol-2-yl)-1,3-dimethylurea. Note, Pentachlorophenol, 2,4,6-trichlorophenol and carbendazim also had very high hazard for acute aquatic toxicity and were only shown once in part A.

Chemical annotations with Level 1 or Level 2 confidence that were classified as having very high hazard potential for carcinogenicity and acute aquatic toxicity—based on authoritative sources in the EPA Cheminformatics module—are plotted according to their relative peak area abundance in Figure 4. Although peak area is not suitable for absolute quantitation, it provides a useful basis for comparing the relative abundance of analytes across sites. Six compounds met these criteria for being potential carcinogens: three pesticides (pentachlorophenol, 2,4,6-trichlorophenol, and carbendazim), one organohalogen flame retardant (tris(2-chloroethyl) phosphate), one artificial sweetener (saccharin), and one industrial chemical commonly found in plastics, paints, and resins (diethanolamine) (Figure 4A). All compounds identified as potential carcinogens were most abundant at Site 4B, relative to the other sites. These results indicate a concentrated input of hazardous substances at Site 4B.

Chemicals with a very high hazard likelihood for acute aquatic toxicity were detected at varying abundances across different sites (Figure 4B). For example, 8-hydroxyquinoline showed the highest peak area at Site 2. This industrial chemical, commonly used in personal care products as a stabilizer and corrosion inhibitor, was most abundant at Site 4A, followed by Site 5. This pattern may suggest a point-source release near Site 4, with downstream dilution observed at Site 5. Tebuconazole, a fungicide, was most prevalent at Site 4B and is likely linked to wastewater discharge in that area. Dibutyl phthalate (DBP), a widely used plasticizer, was most abundant at Site 3, where physical evidence of plastic pipe debris along the shoreline was also observed. Tebuthiuron, an herbicide commonly used in rangeland and near industrial facilities, showed the highest abundance at Sites 4B and 2 [43]. This herbicide’s presence at Site 4B may be attributed to sewage inputs, while the occurrence at Site 2 could stem from agricultural runoff.

While many of these proposed acute aquatic toxicants are not currently regulated for monitoring in surface waters, some are. Notably, four structurally annotated chemicals—4-nitrophenol, 2,4,6-trichlorophenol, pentachlorophenol, and DBP—are listed among the 126 Priority Pollutants under the National Pollutant Discharge Elimination System (NPDES), established by the Clean Water Act and regulated by the EPA [44]. Given the FBR’s history of flooding, persistent aquatic toxicants may be remobilized. Such remobilization may elevate toxicity exposure risks for aquatic organisms in the region [45,46].

### 3.4. Spatial Distribution of Key Contaminant Classes

The integration of NTA with cheminformatics-based hazard screening offers a valuable strategy for identifying and prioritizing chemicals of concern for future monitoring and regulatory attention [47]. The 20 annotated Level 1 or Level 2 compounds with greatest abundances are listed in Table 1. The dominant compound was dodecyl trimethylammonium, a quaternary ammonium salt (QAS) widely employed in disinfectants, sanitizers, shampoos, and other personal-care products. The pronounced peak at Site 4A, followed by a sharp decline downstream at Site 5, which may suggest a localized source near Site 4 and demonstrates the need for additional evaluation of these sites to better understand sources and spatial differences. The cheminformatic hazard assessment showed that the identified QAS lacked hazard testing for 65% of the endpoints and the majority had inconclusive results. However, QASs are increasingly recognized as emerging contaminants because of their extensive use, incomplete removal in conventional wastewater-treatment plants, and consequent discharge to surface waters [48]. QASs, as a class, have been recognized as potentially toxic to aquatic organisms and promote antibiotic-resistant bacteria in aquatic matrices [49,50]. These findings underscore the need for further toxicological evaluation of dodecyl trimethylammonium. Moreover, this class of chemicals has not been commonly investigated in surface waters following hurricanes, highlighting its novelty in this context (SI Table S12).

Hierarchical clustering analysis (HCA) and principal component analysis plots (PCA) were generated in Compound Discoverer (SI Figures S2 and S3). HCA and PCA were used to evaluate temporal variation across sampled sites based on feature detection and peak-area abundance. Results revealed a small cluster of features contributed to Site 3 and Site 4B being significantly different from the other sampled sites. Additional wastewater tracers with Level 1 and 2 annotation, caffeine, paraxanthine, saccharin, acesulfame, and the *E. coli* metabolite 4-pyridoxic acid, were likewise highest at Site 4. The Cheminformatic hazard profiles of these compounds were relatively low hazard for a majority of the endpoints. However, the presence of these wastewater tracers suggests that additional potential hazards could be present within these sampled locations. Site 4 also showed the greatest peak area abundances of pesticides, pharmaceuticals, PFASs, and other wastewater indicators. Several of these pharmaceuticals were ranked as having medium to very high hazard potential for endocrine disruption, acute aquatic toxicity and acute mammalian oral toxicity. Signs of wastewater input from increased anthropogenic contaminants are consistent with elevated total coliform and *E. coli* counts (9700/200 CFU 100 mL^−1^ at Site 4A; 4100/200 CFU 100 mL^−1^ at Site 4B; see SI Table S3). Field observations confirmed a wastewater-treatment facility adjacent to this reach, which experienced severe flooding. Resulting plant impairment and overflow likely account for the observed chemical and microbial signatures. Co-occurrence patterns for both microbial and chemical contaminants has been commonly observed as correlated in studies [51,52]. Although this study lacks sufficient replicates to draw a statistical inference between these two signatures, the detection of both chemical and microbial markers underscores the need for integrated monitoring in future investigations. Collectively, these findings identify Site 4 as a potential hotspot of co-occurring chemical and pathogen exposure that warrants priority monitoring and remediation.

Notably, the majority of the 20 high-abundance compounds listed in Table 1 were polymer-associated chemicals, with peak levels observed at Sites 3 and 4B. Site 3 exhibited particularly high concentrations of polyethylene glycol (PEG) derivatives (n4–n8). Although PEG compounds are generally considered to pose low hazard, their detection in tandem with elevated levels of dibutyl phthalate (DBP, Figure 4) reinforces their role as indicators of plastic pollution. These results were further supported by the substantial plastic debris (e.g., construction pipes) that was observed during sampling. These findings suggest that polymer-associated chemicals, particularly PEGs, may serve as effective chemical tracers for identifying and monitoring plastic-associated contamination in freshwater systems.

It is important to note that although identification of potentially hazardous compounds can provide insight into the potential for human harm, it does not equate to actual environmental risk. Quantitation of these high-level confidence compounds would be necessary to assess environmental concentration and associated risks of exposure for wildlife and human health. Nevertheless, the combination of non-targeted analysis with hazard classifications for exploring preliminary samples demonstrate their utility in identifying specific chemicals that warrant additional long-term monitoring in the FBR.

### 3.5. Limitations and Contextual Considerations

The limitations of this study include the small number of samples collected and the absence of replicates for statistical analysis. Because hurricane-like weather events are highly improbable for a water body such as FBR, sampling was conducted reactively, leading to resource constraints and challenges in collection. Future natural disaster studies should incorporate replicate sampling across multiple sites to enable quality control, stronger statistical analysis and better identification of emerging contaminant hotspots.

This study was limited by the absence of pre-storm baseline data, which prevented direct comparisons of water quality conditions before and after the hurricane. Future approaches to identify priority sites for baseline profiling could include the use of simulated flood impact models informed by historical storm tracks, projected sea level rise, and hundred-year floodplain maps. We recommend sampling during the season preceding hurricane activity in regions that are frequently impacted and initiating sampling in less hurricane-prone areas when storms are forecasted. While non-targeted analysis can provide an initial hazard profile, longitudinal monitoring of identified hotspots is necessary to assess the persistence of contaminants and to guide more targeted analyses of chemicals of concern.

Additionally, the sample preparation methodology was optimized for the concentration of anionic PFASs, which may have limited the retention or recovery of other polar organic contaminants of emerging concern. Matrix effects and compound-specific losses during extraction and analysis may have led to underrepresentation of certain chemical classes in the final dataset. To achieve a more comprehensive assessment, future studies should incorporate additional samples extracted within two days of collection and apply alternative solid-phase extraction techniques (e.g., polymeric reversed-phase sorbents) designed to capture a complementary range of analytes with shorter half-lives. Despite these constraints, this preliminary analysis offers important insight into PFAS occurrence in the FBR, identifies emerging contaminants of concern, and establishes a foundation for future monitoring efforts. Continued sampling will be essential to track the river’s long-term recovery and to evaluate potential risks to both ecosystem integrity and human health.

## 4. Conclusions

This study presents an exploratory hazard profiling strategy for assessing hurricane impact on a freshwater ecosystem. A multi-tiered analytical approach was taken to integrate non-targeted high-resolution mass spectrometry, suspect screening, targeted analysis, and cheminformatics-based hazard evaluations. This strategy enabled characterization of widespread contamination along the French Broad River after Hurricane Helene. Preliminary hazard profiling revealed that most tentatively annotated compounds pose acute aquatic toxicity risks, while a smaller proportion were linked to human health hazards. Site-specific signatures were detected, specifically plastic-associated chemicals likely due to visible plastic debris and several hazardous substances linked to wastewater infrastructure failure, including PFAS concentrations exceeding EPA drinking-water MCLs. Recommendations for future disaster response contaminant screening studies would be to collect additional sampling replicates and pre-storm baseline data to further validate the identified emerging contaminant hotspots. Findings from this work can serve as foundational data on specific chemicals to monitor and locations to pursue further. This study can inform monitoring, public policies and regulations focused on FBR recovery in response to Hurricane Helene. Collectively, this framework offers scalable potential for broader disaster-response monitoring efforts and underscores the importance of continued chemical and microbial surveillance to safeguard vulnerable freshwater ecosystems and public health.

## Figures and Tables

**Table 1:** Top 20 compounds with the highest peak area abundance and Level 1 or Level 2 annotation confidence and hazard information. Product use categories include personal care products (PCPs), polymer-associated chemicals (PA), flame retardants (FRs), pesticides (PEs), natural products (NPs), and wastewater tracers (WTs). The accompanying red-to-white-to-blue heatmap displays relative peak area abundance across Sites 1–5. Peak-area abundance across sample sites for each compound was color coded using conditional formatting in Excel. Each compound’s minimum and maximum peak-area values across all sites were used to determine the range. The highest peak-area value was shown in red (Max Area column), and the lowest in blue, with a linear gradient interpolated proportionally between the set minimum and maximum, transitioning through white. Hazard classifications include acute mammalian oral toxicity (AMT Oral), carcinogenicity (Carcin), developmental toxicity (Dev), and acute aquatic toxicity (AAT), with levels denoted as low (L), medium (M), high (H), very high (VH), or inconclusive (I). Font styles indicate hazard source: bold for authoritative data, italics for QSAR modeling, and regular font for screening-level data.

#	Name [Common Name or Abbreviation]	PU	CASRN	Formula	Max Peak Area Abund.	Site 1	Site 2	Site 3	Site 4A	Site 4B	Site 5	AMT Oral	Carc	Dev	AAT
1	Dodecyltrimethylammonium	PCP	10182-91-9	C15 H33 N	1.20 × 10^9^							*I*		*L*	*I*
2	(Z)-octadec-9-enamide [Oleamide]	PA	301-02-0	C18 H35 N O	1.14 × 10^9^							L	I	*H*	H
3	Pentaethylene glycol [PEG n5]	PA	4792-15-8	C10 H22 O6	5.33 × 10^8^							L		*L*	L
4	Hexaethylene glycol [PEG n6]	PA	2615-15-8	C12 H26 O7	5.01 × 10^8^							L		*L*	*L*
5	Heptaethylene glycol [PEG n7]	PA	5617-32-3	C14 H30 O8	4.38 × 10^8^							*L*		*L*	*L*
6	Octaethylene glycol [PEG n8]	PA	5117-19-1	C16 H34 O9	3.46 × 10^8^							*L*		*L*	*L*
7	Tris(1-chloro-2-propanyl) phosphate	FR	13674-84-5	C9 H18 Cl3 O4 P	2.63 × 10^8^							M	I	M	M
8	4-Dodecylbenzenesulfonic acid	PCP	121-65-3	C18 H30 O3 S	2.63 × 10^8^							M		*H*	L
9	Xylenesulfonate	PCP	25241-16-1	C8 H10 O3 S	2.43 × 10^8^							*L*		*H*	*M*
10	Diethyltoluamide (DEET)	PE	134-62-3	C12 H17 N O	1.92 × 10^8^							**M**	I	M	**M**
11	N-Methyldioctylamine	PCP	4455-26-9	C17 H37 N	1.87 × 10^8^							*M*		*L*	H
12	2-Naphthalenesulfonic acid	PA	120-18-3	C10 H8 O3 S	1.49 × 10^8^							M		*H*	M
13	2-Methoxy-4,6-bis(isopropylamino)triazine [Prometon]	PE	1610-18-0	C10 H19 N5 O	1.41 × 10^8^							M	**I**	H	**M**
14	2,3,4,5,6-pentachlorophenol	PE	87-86-5	C6 H Cl5 O	1.35 × 10^8^							**H**	**VH**	H	**VH**
15	Dodecyl sulfate	PCP	151-41-7	C12 H26 O4 S	1.31 × 10^8^							M			L
16	N,N′-Dicyclohexylurea	NP	2387-23-7	C13 H24 N2 O	1.29 × 10^8^							*L*		*H*	*M*
17	1,3,7-trimethylpurine-2,6-dione [Caffeine]	WT	58-08-2	C8 H10 N4 O2	1.21 × 10^8^							**H**	I	H	**L**
18	azepan-2-one [Caprolactam]	PA	105-60-2	C6 H11 N O	1.17 × 10^8^							**M**	**L**	**L**	L
19	N,N-Dimethyldecylamine N-oxide	PCP	2605-79-0	C12 H27 N O	9.72 × 10^7^							M	I	*L*	H
20	2,4,6-Trichlorophenol	PE	88-06-2	C6 H3 Cl3 O	8.64 × 10^7^							**M**	**VH**	I	**VH**

## Data Availability

The original data presented in the study are openly available in Zenodo at https://doi.org/10.5281/zenodo.17059164.

## Supplementary Materials

The following supporting information can be downloaded at: https://www.mdpi.com/article/10.3390/toxics13110905/s1. References [53,54] are cited in the Supplementary Materials.

## Abbreviations

The following abbreviations are used in this manuscript:

DBP	Dibutyl phthalate
EPA	United States Environmental Protection Agency
FBR	French Broad River
LC-HRMS	Liquid Chromatography–High Resolution Mass Spectrometry
MCL	Maximum contaminant level
PFAS	Per- and polyfluoroalkyl substances
QAS	Quaternary ammonium salt
WNC	Western North Carolina
