# Supplementary material for: Preliminary Identification of PFAS and Other Emerging Contaminants in the French Broad River, NC Post-Hurricane Helene
Source: Toxics. 2025 Oct 22;13(11):905. doi: 10.3390/toxics13110905 (PMC12656598; doi:10.3390/toxics13110905)
Supplement: Supplementary file 1 [file toxics-13-00905-s001.zip › toxics-3886028-supplementary.pdf]

Supplementary Information: Preliminary Identification of PFAS and other Emerging Contaminants in the French Broad River, NC Post-Hurricane Helene

Table S1. Sample Site information.

Site #	Address	Location Key	GPS Coordinates
Site 1	834 Riverside Dr, Asheville, NC 28804	Grainger	35.61123, -82.576467<br>35°35'51", 82°34'35"
Site 2	1050 Riverside Dr, Asheville, NC 28804	Silver Line	35.625429, -82.583083<br>35°37'32", 82°34'59"
Site 3	1420 Riverside Dr, Asheville, NC 28804	French Broad	35.626082, -82.601331<br>35°37'34", 82°36'05"
Site 4A	145 Blannahassett Island Rd, Marshall, NC 28753	Blannahassett Bridge	35.795471, -82.685632<br>35°47'44", 82°41'8"
Site 4B	145 Blannahassett Island Rd, Marshall, NC 28753	Marshall Island Standing water	35.796641, -82.687854<br>35°47'48", 82°41'16"
Site 5	3137 US-25 70 E, Del Rio, TN 37727	Del Rio	35.924541, -83.020886<br>35°53'43", 82°49'26"

Sampling Protocol

At each site, a 250 mL grab sample was taken approximately 3 meters from the right bank, at mid-depth (approximately 0.3 meters) within the main flow of the river, to capture representative conditions. Flow information was not collected at each of the sites but was measured by the United States Geological Survey (USGS) sampling station near Site 1 at an average discharge of 53.8 m^3/s (**Table S2**) [1]. Steps were taken to avoid disturbing the sediment during sampling. Samples were collected using pre-cleaned, acid-washed polypropylene bottles. To avoid cross-contamination, each bottle was rinsed once with ambient river water before final sample collection.

Table S2. Water Quality Parameters from three locations along the French Broad River in close proximity to the same sites samples were generated as a daily mean value on October 12, 2024 from the USGS locations. All data was accessed through: https://waterdata.usgs.gov/.

Descriptors	Location 1	Location 2	Location 3
USGS Location Code	3447890	3451200	3451500
French Broad River Access Point	BLUE RIDGE PKWY AT BENT CREEK NC	HAYWOOD RD AT ASHEVILLE, NC	ASHEVILLE, NC
Lat/Long	35°29'59", 82°35'34"	35°35'06", 82°34'07"	35°36'32", 82°34'41"
Water Temp °C	14.4	14.7	14.8
Dissolved Oxygen (mg/L)	9.5	9.3	9.3
Specific Conductance (uS/cm @25C)	34	37	47
Discharge cubic feet/second	N/A	N/A	1910
Gage Height (ft)	N/A	N/A	2.22

Assessment of *E. coli* and coliform presence

Upon returning to the Appalachian State Ecotoxicology lab, field water samples were tested for presence of *E. coli* and other coliform bacteria with the use of 3M™/Neogen® Petrifilm™ E. coli/Coliform Count (EC) Plates. Samples were shaken gently, and 1mL of sample was immediately transferred to a fresh EC plate. Plates were incubated at 37°C for 48 h and then placed under a low power dissection scope for interpreting and counting colonies. Colony numbers for *E.coli* and total coliform are reported per 100 mL to conform to US EPA benchmarks for drinking water and recreational use (see Table S3).
Protocol: https://www.neogen.com/categories/microbiology/petrifilm-e-coli-coliform-count-plates?utm_medium=redirect&utm_source=vanity-url&utm_campaign=www.3m.com/3M/en_LB/p/d/v000530640/.

Table S3. *E.coli* and total coliform colony numbers from French Broad River surface water samples collected October 12, 2024. Bacteria counts were determined with the use of 3M™ Petrifilm™ E. coli/Coliform Count Plates. US EPA Recreational waters benchmarks: <200/100mL total coliform colonies for swimming, <1000/100mL total coliform colonies for fishing/boating, <2000/100mL for domestic water supply, before treatment. The drinking water standard is less than 1 colony of total coliform / 100 ml with all *E. coli* absent.

Sample Site Number	Distance downstream from River Arts District, Asheville, NC (km)	*E.coli* bacteria colony number (100mL)	Total coliform bacteria colony number (100mL)
Site 1	3.62	200	2800
Site 2	5.03	300	3600
Site 3	8.03	100	2800
Site 4a	34.90	200	9900
Site 4b	35.11	200	4300
Site 5	84.79	100	2400
Field blank	59.85	0	0

Table S4. Chemical Reference standards and reagents used in confirmation and quantitation of analytes. Supplier information included. Reagents include ammonium acetate (HPLC grade, JT Baker), ammonium hydroxide (HPLC grade, ACROS Organics), Sodium Phosphate Dibasic (Fisher Chemical), Sodium Phosphate monobasic (Sigma Aldrich), Methanol (HPLC grade, Birch biotech), formic acid (Fisher Chemical), DI water, water (HPLC grade, Fisher Chemical), acetonitrile (HPLC grade, Fisher Scientific).

Material	Cas No.	Supplier and P/N	Purity/Grade/ Specification	Part Number & Lot No.
Caffeine	58-08-2	Thermo Scientific	99%	A10431.22 Q07I022
Tris(2-chloroethyl) phosphate	115-96-8	Sigma Aldrich	97%	07430JR
25 Native PFAS Primary Standard	N/A	Wellington Laboratories	98%	EPA-533PAR 533PAR0723
PFAS Isotope Performance Standard Mixture (IPS)	N/A	Wellington Laboratories	98%	EPA-533IS 533IS0322
PFAS Isotope Dilution Standard (IDAS)	N/A	Wellington Laboratories	98%	EPA-533ES 533ES0623
Paraben and Phenol mixture	N/A	Cambridge Isotopes Laboratories	98%	ES-5599 PR-34134
Pesticide Mixture	N/A	Restek	<95%	31975 A0198992

Table S5. Tandem MS instrument methods

**Instrument:**	SCIEX 7500 Triple Quad (SCIEX, Framingham MA)
**Software:**	SCIEX OS 3.3.1<br>(SCIEX, Framingham MA)
**Analytical Column:**	Phenomenex Gemini C18, 3µm, 110 Å, 2x50 mm
**Delay Column:**	Phenomenex Gemini C18, 5µm, 110 Å, 3x50 mm
**Gas 1:**	40 psi
**Gas 2:**	70 psi
**Curtain Gas:**	40 psi
**CAD gas:**	10 psi
**Oven Temperature:**	40 °C
**Source Temperature:**	300 °C
**Spray Voltage:**	2500 V
**Ionization Mode**	Negative; Electrospray Ionization
**Injection Volume**	2 µL
**Flow Rate**	0.4 mL/min
**Mobile Phase A**	10 mM Ammonium Acetate in 95:5 Water:Methanol

**Instrument:**	SCIEX 7500 Triple Quad (SCIEX, Framingham MA)
**Mobile Phase B**	10 mM Ammonium Acetate in 95:5 Methanol:Water
**Mobile Phase Gradient**	The gradient was 10% B for 1 min, ramp to 100% B in 6.5 min, hold at 100% B for 2 min. The total run time was 12.5 min.

Table S6. PFAS Quantitation Methods and Isotope Recovery Responses.

Extraction and quantitation methods followed EPA method 533. Quantitation was completed through the liquid chromatography Science ExionLC AC with a triple quadrupole mass spectrometer (Sciex 7500) on a HPLC Column (Phenomenex Gemini C18, 2x50mm, 3 um) with a PFAS delay column (Phenomex Gemini C18, 3x50mm, 5um). Calibration curve included 7 standards ranging 0.25 to 50 ng/mL with four quality control standards at concentration (0.25, 1, 10 and 40 ng/mL). The minimum reporting limit was 0.25 ng/mL for all analytes with the exception of PFBA. The Isotope dilution analogue standard (IDAS) recovery remained between 70-130%, most isotope performance standards (IPS) between 50-150%, and lab fortified blank recovery between 70-130%. There was one IPS failure for Site 4A and the IPS standard for PFBA (38% recovery); however, all other analytes passed in that sample. Raw data and QC information can be found in the supplementary excel file.

IPS Recovery (50-150%)	PFBA	PFPeA	PFHxA	PFHpA	PFOA	PFNA	PFBS	PFBS-2	PFHxS	PFOS	NFDHA
Ave	71.45	71.45	96.84	96.84	96.84	96.84	94.79	94.79	94.79	94.79	96.84
SD	16.80	16.80	2.67	2.67	2.67	2.67	2.69	2.69	2.69	2.69	2.67

IDA % Recovery (50–200%)	PFBA	PFPeA	PFHxA	PFHpA	PFOA	PFNA	PFBS	PFBS-2	PFHxS	PFOS	NFDHA
Ave	84.89	79.71	73.95	78.88	76.96	70.53	60.52	60.52	90.74	91.13	73.95
SD	4.55	18.86	7.55	4.47	6.63	8.84	12.33	12.33	5.61	2.29	7.55

Table S7. High Resolution Mass Spectrometry (HRMS) Methods.

**UPLC Parameters**	
**Data Software**	Xcalibur Version: 4.5.445.18
**Autosampler Temperature**	10 °C
**Column**	Waters (Milford, MA) Acquity UPLC HSS T3 (2.1 × 100 mm, 1.8 µm) column
**Injection Volume**	3 µL
**Column Temperature**	50 °C
**Flow Rate**	0.4 mL/min
**Polarity**	Positive and Negative

**UPLC Parameters**	
**Data Software**	Xcalibur Version: 4.5.445.18
**Autosampler Temperature**	10 °C
**Positive Ion Mobile Phases**	**A**: 0.1% formic acid in water<br>**B**: 0.1% formic acid in methanol
**Negative Ion Mobile Phases**	**A**: 5 mM ammonium acetate in water<br>**B**: Acetonitrile |100%
**Mobile Phase Gradient**	Initial hold at 99% A for 1 min, ramp to 100% B in 15 min, hold 100% B for 3 min, reverse to 99% A in 0.5 min, hold at 99% A for 2.5 min. Divert to waste 0-0.5 min.
**Run Time**	22 mins
**HRMS Parameters**	
**Ionization/Acquisition Mode:**	H-ESI
**Polarity:**	Positive and Negative
**Acquisition Range:**	MS Full scan mode with a range of 100-1000 m/z (60,000 resolution)<br>MS/MS using data dependent acquisition, for the top 20 ions. (30,000 resolution) Scanning 0.5-19.5 min.
**Scan Time:**	1 s
**Capillary Voltage:**	3.5 kV+, 2.5 kV-
**HCD Collision Energy:**	20, 40, 60%
**Ion Transfer Tube Temperature:**	325°C
**Vaporizer Temperature:**	350°C
**Sheath Gas (arb):**	50
**Aux Gas (arb):**	10
**Sweep Gas (arb):**	1

HRMS QA/QC

Sample analysis using the HRMS system, followed by data processing in Thermo Fisher Tracefinder (Milford, MA), was conducted under rigorous QA/QC protocols to ensure data integrity and reproducibility. Sample preparation included blanks, standards, and pooled quality control (QC) samples to monitor consistency throughout the workflow. All samples, controls, and standards were processed in a single batch, with Waters system suitability standards (Milford, MA) injected in triplicate at both the beginning and end of the run. Sample sequence order was randomized to minimize potential bias. Sample pools were injected at the start, midpoint, and conclusion of the run, with sample blanks interspersed to track carryover and background contamination. Instrument calibration involved verification of mass accuracy, resolution, and sensitivity using designated reference compounds. Prior to sample injection, system suitability was confirmed by evaluating the Waters system suitability standards for retention time stability (<0.1 min), peak area precision (RSD <30%), mass accuracy (m/z <5 ppm), and peak shape, as demonstrated in **Table S8**. QC pooled samples were reviewed post-run to assess technical variability and reproducibility. Deviations from expected performance metrics were not observed and thus reinjection and reanalysis was not completed for this sample set. Post-acquisition, Compound

Discoverer software was used to process data with strict alignment, peak picking, and normalization settings.

Table S8. HRMS Suitability Standards Performance of the Waters LCMS QC Reference Standard (SKU: 186006963)

Positive Mode Suitability Standards*

Compound	Acetaminophen		Caffeine		Leucine-Enkephalin		Sulfadimethoxine		Sulfaguanidine	
Area (Ave/ %RSD)	6.94E+08	17%	2.46E+08	20%	2.12E+08	18%	9.04E+08	17%	3.7E+08	19%
RT min (Ave/SEM)	4.01	0.002	6.06	0.002	8.46	0.002	8.15	0	1.37	0.002
Delta Mass Accuracy (ppm) (Ave/SEM)	-1.73	0.03	-1.55	0.08	-1.04	0.06	-1.46	0.07	-1.16	0.05

*Note: LC Suitability standard was injected at the beginning (n= 3) and at the end (n=3) of the run. The last injection #6 was excluded from analysis as injection response did not properly inject.

Negative Mode Suitability Standards**

Compound	Leucine-Enkephalin		Sulfadimethoxine		Val,-Tyr-Val	
Area (Ave/ %RSD)	4.60E+07	2.6%	3.28E+07	4%	3.98E+07	3.8%
RT min (Ave/SEM)	6.005	0.002041	6.165	0.006124	4.456667	0.001925
Delta Mass Accuracy (ppm) (Ave/SEM)	0.264033	0.102732	0.689383	0.089383	0.14885	0.02264

**Note: LC Suitability standard was injected at the beginning (n= 3) and at the end (n=3) of the run.

Post Acquisition

Instrument data was collected using Thermo Fisher Xcalibur (.raw file type) software and processed for suspect screening and non-targeted analysis via Compound Discoverer 3.3 software. Data processing steps included, initial m/z detection, reaction time alignment, signal thresholding and normalization using QC pool standards, spectral deconvolution, peak shape evaluation, and background subtraction (Peak areas must be 5-fold higher than the sample blanks). Features then underwent molecular formula assignment, structural similarity searching. Additional evaluation of the features included screening their precursor mass, predicted molecular formula and/or their MS/MS fragmentation data against compound database, compound lists, and spectral libraries (**Table S9**). Basic statistics of feature peak area evaluations included t-tests and QC peak area evaluation where features with RSD% larger than 30% were not included. Additional statistics and chemometric analysis included differential analysis and hierarchical clustering analysis.

Table S9. List Libraries and Databases used for suspect and nontargeted analysis.

Type	Lists, Libraries, and Databases
**ChemSpider Libraries**	EPA DSSTox (900,000 chemicals)<br>EPA ToxCast (4,400 chemicals)<br>FDA (1616 chemicals)<br>ACToR: Aggregated Computational Toxicology Resource<br>DrugBank<br>EAWAG Biocatalysis/Biodegradation Database
**Mass List**	UpdatedPolymerAdditiveList2021v3 (5616 chemicals)<br>PFAS_NIST (4951 chemicals)<br>EPA_PesticidesList (2481 chemicals)<br>EPA_ContaminantOfEmergingConcern (50,324 chemicals)
**MzVault Spectral Database**	080522 HHEAR POSITIVE and NEGATIVE<br>080522 MSMLS POSITIVE and NEGATIVE<br>080522 NIST 2020 POS and NEG<br>061724_EPAToxCast_POS and Neg<br>050324_RestekPesticides POS and NEG<br>051424_Phthalates POS and NEG<br>051724_PFAS POS and NEG<br>051524_Phenols_POS and NEG<br>051624_FlameRetardants_POS and NEG

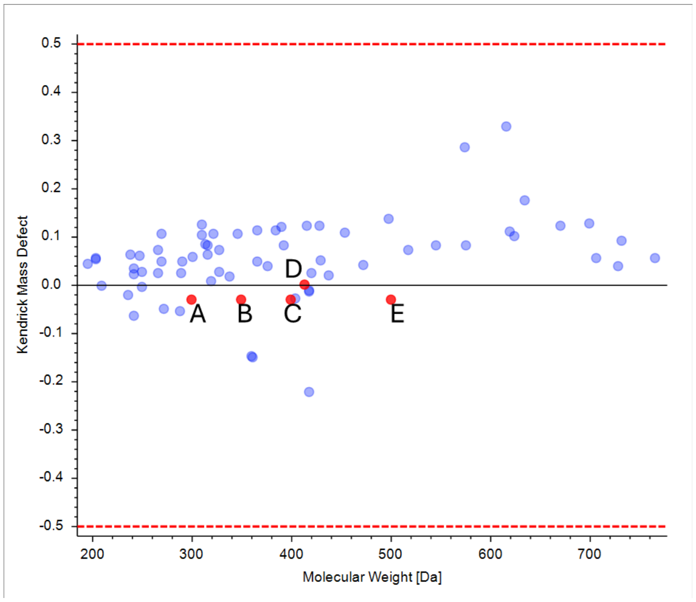

Figure S1. Kendrick Mass Defect (CF2) plotted against molecular weight with notes on specific level 1 annotations including A) Perfluoro-1-butanesulfonic acid (PFBS), B) Perfluoro-1-pentanesulfonate, C) Perfluoro-1-hexanesulfonic acid (PFHxS), D) Perfluorooctanoic acid (PFOA) and E) Perfluoro-6-methylheptanesulfonic acid (PFOS Branched).

Table S10. Feature Details from the sites sampled.

**Feature Details**	**Pos**	**Neg**
Total Features after background subtraction	2228	1033
Filtering Method	372	273
Filtered with Hazard Information	267	201
Filtered with Product Use Information	73	44

Figure S2. Filtration method used to reduce false positives and noise and prioritize higher confidence annotations of environmental contaminants in the sites sampled.

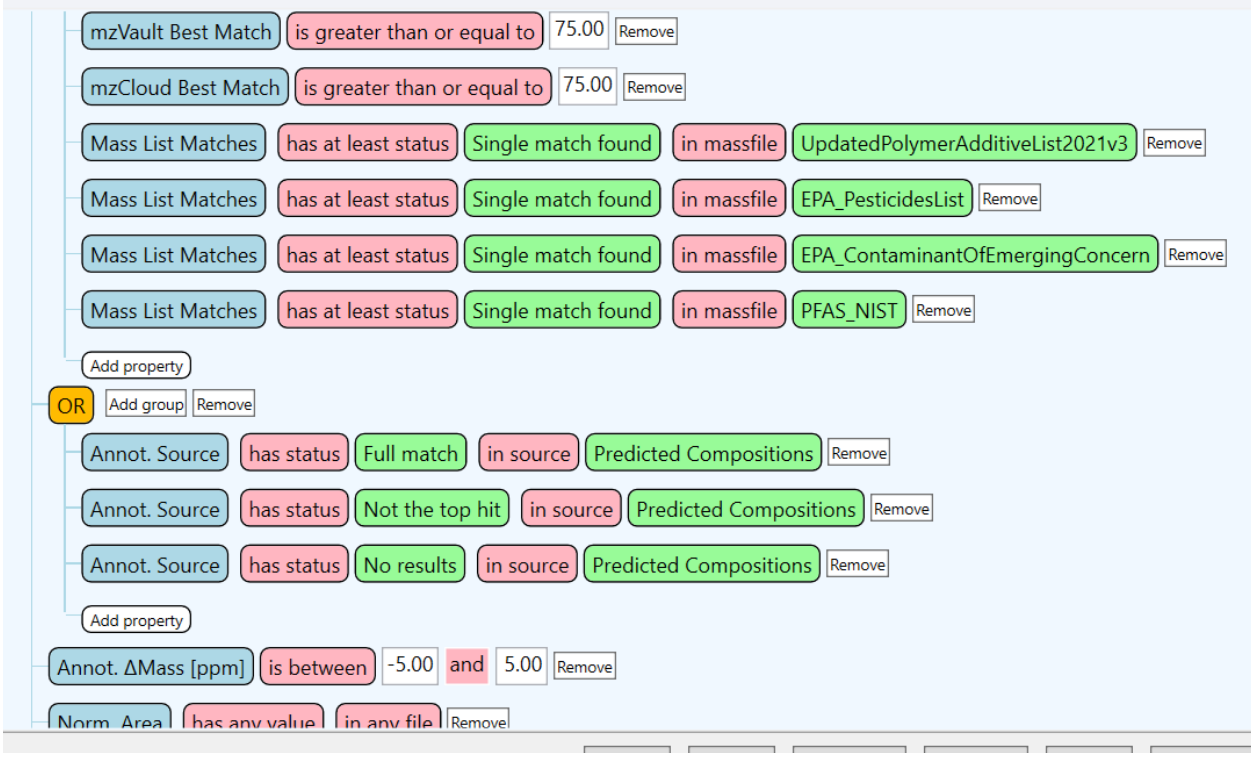

General Hazards for all annotated features

This process identified 468 compounds with available hazard information, as detailed in the SI Excel Data File. More than 60% were classified as having medium to very high hazard potential, particularly for acute aquatic toxicity. For human health hazards, at least 10% of the chemicals that were classified as medium to very high hazard potential included compounds with developmental toxicity (59%), acute mammalian oral toxicity (46%), mutagenic genotoxicity (38%), endocrine disruption (23%), skin irritation (21%), skin sensitization (18%), eye irritation (14%), reproductive toxicity (12%), repeat exposure systemic toxicity (10%), and known or suspected carcinogenic properties (10%). It is important to note that although hazard classification can provide insight into the potential for human harm, it does not equate to actual environmental risk. For instance, 78% of these 468 compound assignments were matched to the mass list as Level 4 annotations, indicating that there is a low level of confidence of the assignments. Due to the low degree of confidence, further experiments with chemical reference standards are necessary to confirm their identity and the concentration present, which can contribute to true estimates of human health risk. The majority of annotated compounds lacked quantitation data necessary to accurately assess environmental concentrations and associated risks.

Table S11. Selected features from chemicals classes including Plastic Additives, Pesticide/Herbicide, Wastewater Tracers and Pharmaceuticals that were structurally annotated via spectral library score greater than 75% (Confidence (Conf.) Level 2) or by compound confirmation using reference standards retention time (RT), MS, and MS/MS (Conf. level 1). Labeled Acronyms: polarity(Pol, positive (+), negative (-)), Acute Mammalian Toxicity Oral (AMT Oral), and Acute Aquatic Toxicity (AAT), Endocrine Disrupting Chemical (EDC). Hazard levels denoted as low (L), medium (M), high (H), very high (VH), or inconclusive (I). Hazard sources are indicated by font style: **bold** for authoritative data, *italic* for QSAR modeling, and regular font for screening-level data. The accompanying red to white to blue heatmap displays relative peak area abundance across Sites 1–5. Color coding of peak area abundance across sample sites for each compound was performed using conditional formatting in Excel. The minimum and maximum peak area values for each compound were evaluated across all locations to determine the range. The highest numerical peak area value was represented in red and the lowest in blue, with a linear gradient interpolated proportionally between the set minimum and maximum, transitioning through white.

Name	Conf.	CAS	Formula	Spectral Library Score	RT [min]	Pol	Area Max	AMT Oral	AAT	EDC	Site 1	Site 2	Site 3	Site 4A	Site 4B	Site 5
**Plastic Additive**																
4,4'-Dihydroxydiphenylsulfone (BPS)	1	80-09-1	C12 H10 O4 S	95.6/94.2	7.5	+	3.10E+07	M	M	H						
4-Nitrophenol	1	100-02-7	C6 H5 N O3	98.9/99.8	6.2	-	9.19E+06	**M**	**H**	H						
Tris(2-chloroethyl) phosphate	1	115-96-8	C6 H12 Cl3 O4 P	97.2/83.7	9.9	+	7.22E+07	**M**	L							
2,5-di-tert-Butylhydroquinone	2	88-58-4	C14 H22 O2	93.5	7.9	-	1.32E+07	M	H							
Dibutyl phthalate	2	84-74-2	C16 H22 O4	95.1/68.2	8.5	-	5.52E+06	L	**VH**	**H**						
N,N'-Diphenylguanidine	2	102-06-7	C13 H13 N3	97.8/89.2	6.3	+	6.68E+07	**M**	H							
Citroflex 4	2	77-94-1	C18 H32 O7	77.2/76.4	14.6	+	1.15E+07	*L*	*H*	*L*						
2-Naphthalenesulfonic acid	2	120-18-3	C10 H8 O3 S	88.1	5.2	-	1.49E+08	M	M	*L*						
Tris(1-chloro-2-propanyl) phosphate	2	13674-84-5	C9 H18 Cl3 O4 P	97.1/92.3	12.4	+	2.63E+08	M	M	H						
Caprolactam	2	105-60-2	C6 H11 N O	98.6/84.6	7.4	+	1.17E+08	**M**	L	*L*						
**Pesticide/Herbicide**																
Carbendazim	1	10605-21-7	C9 H9 N3 O2	95/99.9	5.3	+	3.47E+07	L	**VH**	H						
Prometon	1	1610-18-0	C10 H19 N5 O	99.8/95	9.7	+	1.41E+08	M	**M**	L						
Tebuconazole	1	107534-96-3	C16 H22 Cl N3 O	91.4/80.3	13.6	+	7.14E+06	**M**	**VH**	H						
Tebuthiuron	1	34014-18-1	C9 H16 N4 O S	72.1/76.4	10.1	+	4.09E+06	**M**	**VH**							

Supplementary Information: Preliminary Identification of PFAS and other Emerging Contaminants in the French Broad River, NC Post-Hurricane Helene

Name	Conf.	CAS	Formula	Spectral Library Score	RT [min]	Pol	Area Max	AMT Oral	AAT	EDC	Site 1	Site 2	Site 3	Site 4A	Site 4B	Site 5
DEET	1	134-62-3	C12 H17 N O	98.9	11.3	+	1.92E+08	**M**	**M**							
2,4,6-Trichlorophenol	2	88-06-2	C6 H3 Cl3 O	/88.6	6.7	-	8.64E+07	**M**	**VH**	H						
2-Hydroxyatrazine	2	2163-68-0	C8 H15 N5 O	95	6.1	+	2.17E+07	*M*	*L*	*L*						
Pentachlorophenol	2	87-86-5	C6 H Cl5 O	100	8.4	-	1.35E+08	**H**	**VH**	H						
Imazapyr	2	81334-34-1	C13 H15 N3 O3	92.6/86.4	7.2	+	2.31E+07	L	**L**	*L*						
Metolachlor ESA	2	171118-09-5	C15 H23 N O5 S	88.4	6.9	-	3.56E+06	*L*	*M*	*H*						
**Wastewater Tracer**																
Caffeine	1	58-08-2	C8 H10 N4 O2	99.1/81.1	6.1	+	1.21E+08	**H**	**L**	H						
Paraxanthine	2	611-59-6	C7 H8 N4 O2	96	4.9	+	1.39E+07	*M*	*L*	*L*						
Saccharin	2	81-07-2	C7 H5 N O3 S	99.8	3.2	-	1.75E+07	L	L	*L*						
Acesulfame	2	33665-90-6	C4 H5 N O4 S	98	1.6	-	1.93E+07	*M*	*L*	*L*						
4-Pyridoxic acid	2	82-82-6	C8 H9 N O4	77/92.6	3.4	-	1.34E+07	L	*L*	*L*						
**Pharmaceutical**																
Carbamazepine	2	298-46-4	C15 H12 N2 O	89.1/83	8.9	+	2.07E+07	M	**M**	*L*						
Cetirizine	2	83881-51-0	C21 H25 Cl N2 O3	93.9	11.1	+	1.17E+07									
Amfepramone	2	90-84-6	C13 H19 N O	78.2	6.7	+	3.58E+07	H	*M*	*L*						
Atenolol acid	2	N/A	C14 H21 N O4	98.5	5.4	+	4.50E+07									
Benzoic acid	2	65-85-0	C7 H6 O2	99.7	1.8	-	1.51E+07	M	M	H						
Fexofenadine	2	83799-24-0	C32 H39 N O4	99.1	10.4	+	6.23E+07	*M*	*VH*	*H*						
Florfenicol	2	73231-34-2	C12 H14 Cl2 F N O4 S	87.8	6.6	-	6.70E+06	*L*	*H*	*L*						
Benzoylecgonine	2	519-09-5	C16 H19 N O4	97.9	6.6	+	3.45E+07	*M*	*H*	*H*						
O-Desmethyl-cis-tramadol	2	80456-81-1	C15 H23 N O2	75.9	5.4	+	1.57E+07	*M*	*M*	*H*						
O-Desmethylvenlafaxine	2	93413-62-8	C16 H25 N O2	90.7	6.6	+	2.66E+07	*M*	*H*	*H*						
Dextrorphan	2	125-73-5	C17 H23 N O	99.3	6.8	+	7.32E+06									
Metoprolol	2	51384-51-1	C15 H25 N O3	99/86.5	7.1	+	1.90E+07	M	**H**	*L*						
Myristyl sulfate	2	4754-44-3	C14 H30 O4 S	97.4	11.4	-	3.84E+07									
Lamotrigine	2	84057-84-1	C9 H7 Cl2 N5	99.9	6.8	+	5.83E+07	H	*H*	*L*						
Lidocaine	2	137-58-6	C14 H22 N2 O	92.8	5.9	+	1.74E+07	H	L	*L*						

Supplementary Information: Preliminary Identification of PFAS and other Emerging Contaminants in the French Broad River, NC Post-Hurricane Helene

Name	Conf.	CAS	Formula	Spectral Library Score	RT [min]	Pol	Area Max	AMT Oral	AAT	EDC	Site 1	Site 2	Site 3	Site 4A	Site 4B	Site 5
Losartan	2	114798-26-4	C22 H23 Cl N6 O	94.9	11.5	+	2.47E+07									
Methocarbamol	2	532-03-6	C11 H15 N O5	97.4/87.5	7.6	+	8.18E+06	M	L	*L*						
Irbesartan	2	138402-11-6	C25 H28 N6 O	77	12.0	+	8.76E+06									
Topiramate	2	97240-79-4	C12 H21 N O8 S	88	7.7	-	2.88E+06	*M*	*M*	*L*						
Valsartan	2	137862-53-4	C24 H29 N5 O3	97.6	12.6	+	1.79E+07	*I*	*I*	*H*						

Table S12. Summary of Contamination Related Studies Post Hurricanes within the US.

Ref.	Disaster (Year)	Time Post Storm	Location	Sample Type	Contaminants Targeted	Trends
Martinez et al. (2022)[2]	Hurricane Dorian (2019)	2 days and 6 months	East coast of Florida	Surface water: time-series sampling before/during/after storm	51 PFAS	Sum total pfas during storm was 4.69 ± 1.70 ng/L and 2 days post storm were 4.69 ± 1.70 ng/L compared to 2.36 ± 0.96 six months post storm. PFOS nearly doubled in concentration during storm and returned to baseline within 2 days. Other PFAS showed little/no change.
Lin et al. (2020)[3]	Hurricanes Maria (2017)	3 to 5 months	Puerto Rico	drinking water: pre/post sampling of municipal & private wells	inorganic (18 trace elements) and organic trace pollutants (200 micropollutants)	Compounds elevated post storm: Arsenic, sucralose, perfluorooctanoic acid (PFOA, 2.0 ng/L), atrazine-2-hydroxy, benzotriazole, acesulfame, and prometon.
Hedgespeth et. al.(2021)[4]	Hurricane Florence (2018)	10 days to 3 months	Coastal North Carolina	Pre/post sampling waste, ground and surface water.	Suspect Screening	PFAS, 4000 unique features. Spatial differences in greater dynamic response of relative abundance of industrial/manufactured chemicals, drugs, pesticides/antimicrobials, and natural products detected
Fisher et al (2016)[5]	Hurricane Sandy (2012)	1 Year	Hempstead Bay, New York	Pre/post storm bed sediment (2010 vs 2013)	74 wastewater tracers and steroid hormones, and total organic carbon	PAH frequent detection

Figure S3. Principle Component Analysis plots along with their loadings plots demonstrates strong differences in site 3 and 4B from other sites sampled.

Polarity	Graph
Positive	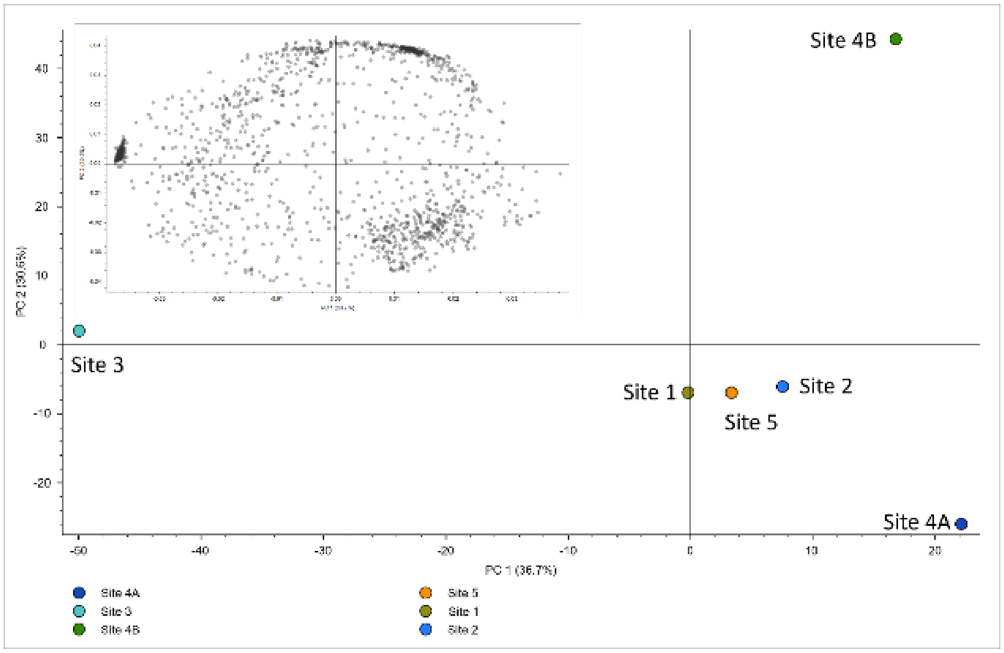
Negative	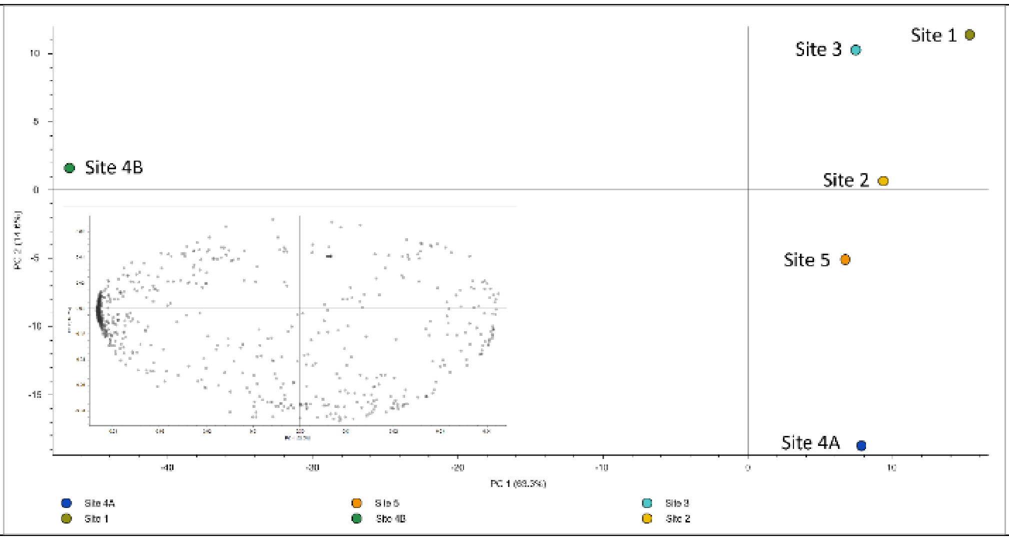

Figure S4. Hierarchical clustering heatmap of features present in all 5 sample sites.

Polarity	Figure
Positive	(see below)
Negative	(see below)

Positive

Positive	Key
F46	Field Blank
F37	Site 4A
F45	Site 5
F47	Site 3
F48	Site 1
F53	Site 4B
F57	Site 2
F54	Pool1
F55	Pool2
F56	Pool3

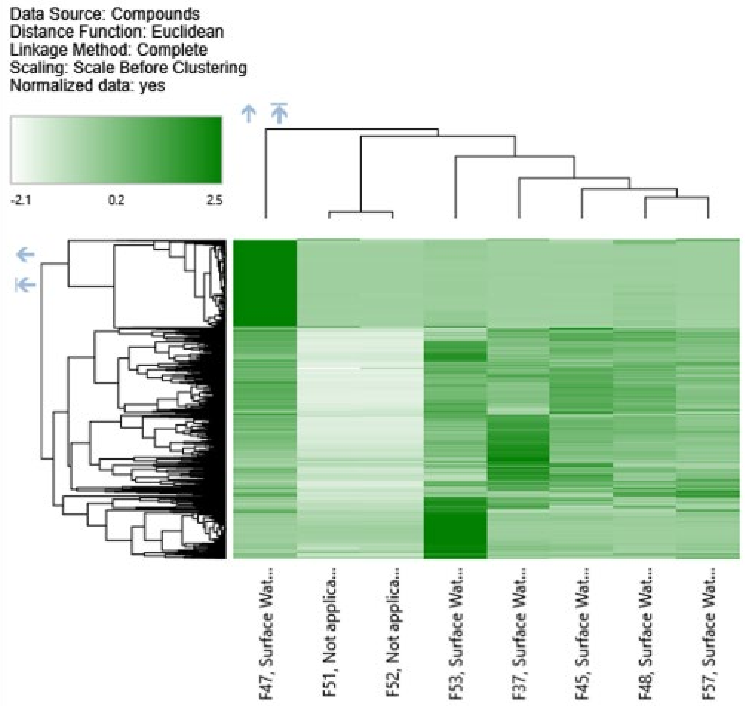

Negative

Negative	Key
F36	Site 4A
F37	Site 5
F38	Field Blank
F39	Site 3
F40	Site 1
F43	Site 4B
F48	Site 2
F45	Pool1
F46	Pool2
F47	Pool3

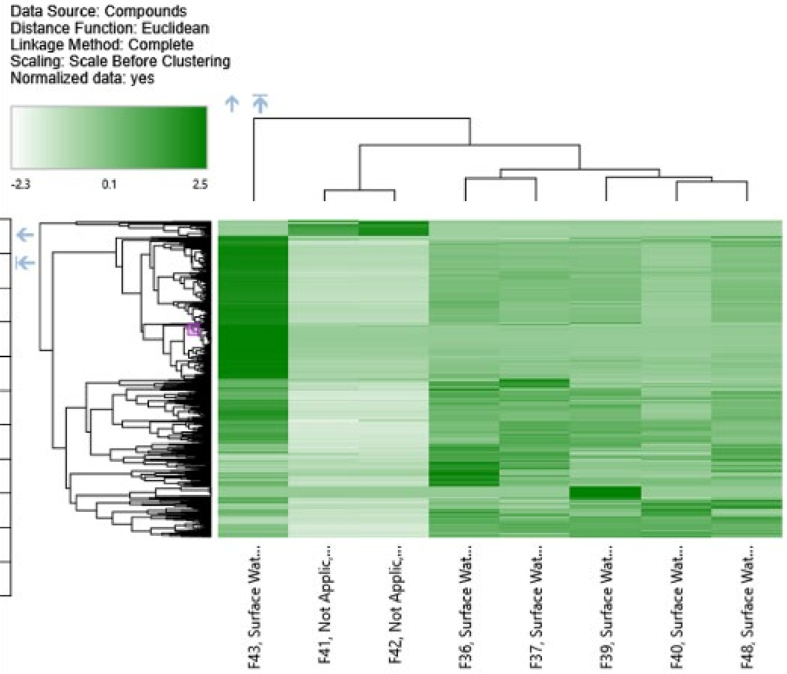

Figure S5. PFBS original Quant parameters site 4B using confirmation ion 79.9.

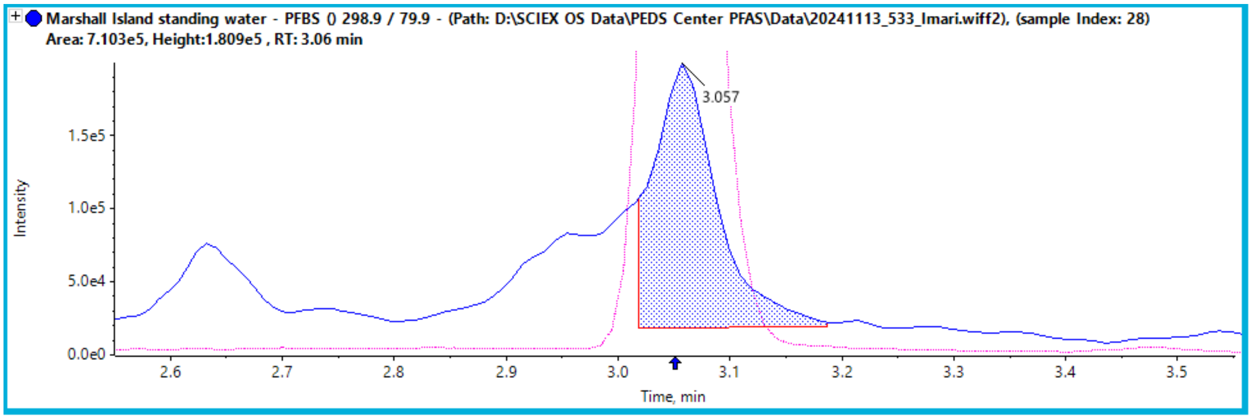

PFBS 2 quant values in site 4B with readjusted confirmation ion (98.9).

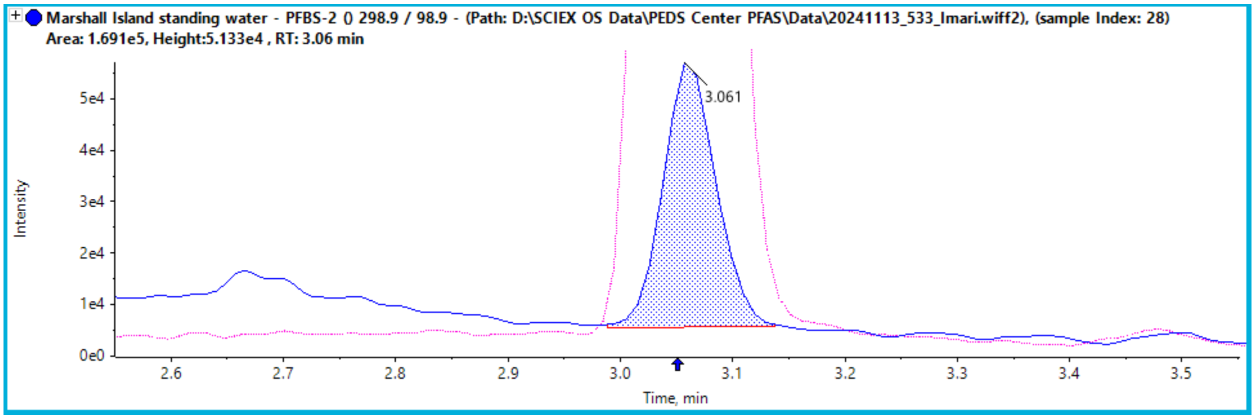

Figure S6. Total ion chromatogram of the 6 pooled water samples injected in positive ionization (Top) and negative ionization (Bottom).

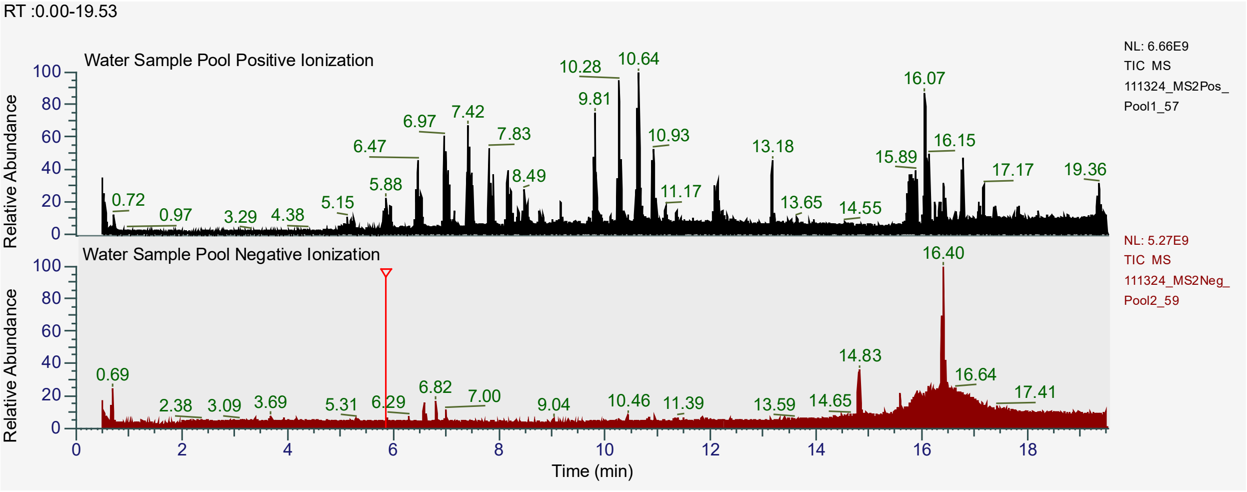

Figure S7. Precursor ion m/z plotted by Retention time. Features with MS2 data available are colored in blue.

Positive Mode	Negative Mode
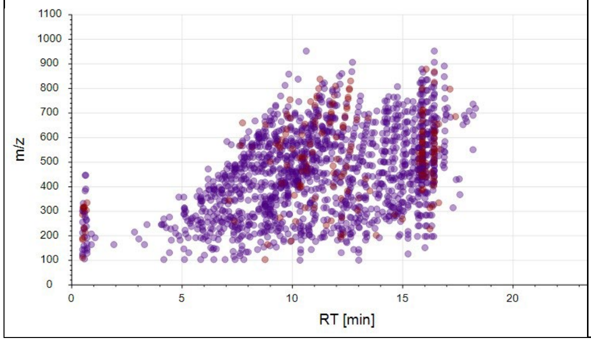	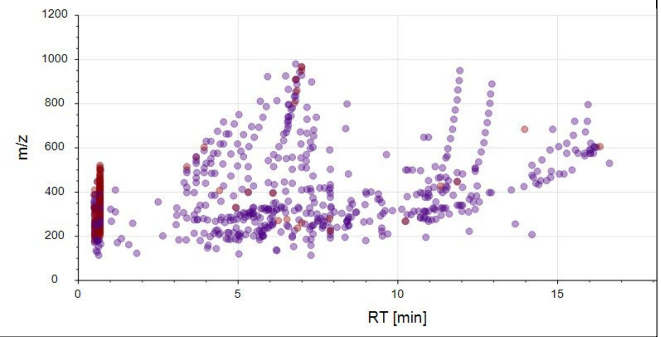

Figure S8 Image of Sample Site 2 inundated with plastic pipe materials on October 12, 2024. Sediment sampling was completed at the time of this photo.

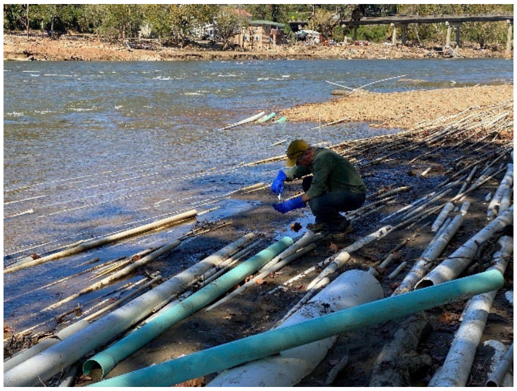

SI Part 1. Data processing workflow information Example for Gap Filled workflow.
All samples were run with and without gap filling to determine unique feature present by site. Positive and negative ionization data files were also processed separately.

Search name: 12272024_WesternNC_NOWELLS_POS_v7_gapfillmzvault
Search description: Untargeted environmental research ID workflow with statistics: Detect and identify unknown compounds with differential analysis.
- Performs retention time alignment, unknown compound detection, and compound grouping across all samples. Predicts elemental compositions for all compounds, and hides chemical background (using Blank samples). Identifies compounds using mzCloud (ddMS2 and/or DIA), ChemSpider (exact mass or formula) and local database searches against Mass Lists (exact mass with or without RT). Performs spectral similarity search against mzCloud for compounds with ddMS2. Applies mzLogic to rank order structure candidates from ChemSpider and mass list matches. Applies spectral distance scoring to mass list and ChemSpider matches. Generates mass defect values in the Compounds table based on selected mass defect type (Kendrick for identifying polymers). Fill gaps by redetecting the peaks by the Fill Gaps node. Performs differential analysis on detected compounds.

Search date: 1/10/2025 10:23:18 PM
Created with Discoverer version: 3.3.3.200

[Input Files (0)]
-->Select Spectra (38)

[Select Spectra (38)]
-->Align Retention Times (54)

[Align Retention Times (54)]
-->Detect Compounds (53)

[Detect Compounds (53)]
-->Group Compounds (25)
-->Merge Features (14)

[Group Compounds (25)]
-->Fill Gaps (50)
-->Calculate Mass Defect (46)
-->Assign Compound Annotations (40)
-->Search mzCloud (51)
-->Search mzVault (56)
-->Predict Compositions (37)
-->Search ChemSpider (22)
-->Search Mass Lists (39)

[Fill Gaps (50)]
-->Apply SERRF QC Correction (55)

[Apply SERRF QC Correction (55)]
-->Mark Background (43)

[Search ChemSpider (22)]
-->Apply Spectral Distance (44)

[Search Mass Lists (39)]
-->Apply Spectral Distance (44)

[Mark Background (43)]

[Calculate Mass Defect (46)]

[Assign Compound Annotations (40)]

[Search mzCloud (51)]

[Search mzVault (56)]

[Predict Compositions (37)]

[Apply Spectral Distance (44)]

[Merge Features (14)]

[Differential Analysis (49)]

Processing node 0: Input Files

Input Data:
- File Name(s) (Hidden):
E:\Orbitrap Raw Data Files\Exploris Raw Files\111324_WesternNCWater_MSPos\111324_Methanol_MSPos_20.raw
E:\Orbitrap Raw Data Files\Exploris Raw Files\111324_WesternNCWater_MSPos\111324_Methanol_MSPos_31.raw
E:\Orbitrap Raw Data Files\Exploris Raw Files\111324_WesternNCWater_MSPos\111324_Methanol_MSPos_38.raw
E:\Orbitrap Raw Data Files\Exploris Raw Files\111324_WesternNCWater_MSPos\111324_Methanol_MSPos_55.raw
E:\Orbitrap Raw Data Files\Exploris Raw Files\111324_WesternNCWater_MSPos\111324_Methanol_MSPos_62.raw
E:\Orbitrap Raw Data Files\Exploris Raw Files\111324_WesternNCWater_MSPos\111324_MS2Pos_BlannahassettBridge_46.raw
E:\Orbitrap Raw Data Files\Exploris Raw Files\111324_WesternNCWater_MSPos\111324_MS2Pos_C7_19.raw

E:\Orbitrap Raw Data Files\Exploris Raw Files\111324_WesternNCWater_MSPos\111324_MS2Pos_DelRio Site 6_50.raw
E:\Orbitrap Raw Data Files\Exploris Raw Files\111324_WesternNCWater_MSPos\111324_MS2Pos_Field Blank_24.raw
E:\Orbitrap Raw Data Files\Exploris Raw Files\111324_WesternNCWater_MSPos\111324_MS2Pos_French Broad Site 3_40.raw
E:\Orbitrap Raw Data Files\Exploris Raw Files\111324_WesternNCWater_MSPos\111324_MS2Pos_Grainger Site 1_48.raw
E:\Orbitrap Raw Data Files\Exploris Raw Files\111324_WesternNCWater_MSPos\111324_MS2Pos_LRB_22.raw
E:\Orbitrap Raw Data Files\Exploris Raw Files\111324_WesternNCWater_MSPos\111324_MS2Pos_LRB_52.raw
E:\Orbitrap Raw Data Files\Exploris Raw Files\111324_WesternNCWater_MSPos\111324_MS2Pos_Marshall Island Standing Water_42.raw
E:\Orbitrap Raw Data Files\Exploris Raw Files\111324_WesternNCWater_MSPos\111324_MS2Pos_Pool1_57.raw
E:\Orbitrap Raw Data Files\Exploris Raw Files\111324_WesternNCWater_MSPos\111324_MS2Pos_Pool2_59.raw
E:\Orbitrap Raw Data Files\Exploris Raw Files\111324_WesternNCWater_MSPos\111324_MS2Pos_Pool3_61.raw
E:\Orbitrap Raw Data Files\Exploris Raw Files\111324_WesternNCWater_MSPos\111324_MS2Pos_Silver Line_44.raw
E:\Orbitrap Raw Data Files\Exploris Raw Files\111324_WesternNCWater_MSPos\111324_MSPos_BlannahassettBridge_45.raw
E:\Orbitrap Raw Data Files\Exploris Raw Files\111324_WesternNCWater_MSPos\111324_MSPos_DelRio Site 6_49.raw
E:\Orbitrap Raw Data Files\Exploris Raw Files\111324_WesternNCWater_MSPos\111324_MSPos_Field Blank_23.raw
E:\Orbitrap Raw Data Files\Exploris Raw Files\111324_WesternNCWater_MSPos\111324_MSPos_French Broad Site 3_39.raw
E:\Orbitrap Raw Data Files\Exploris Raw Files\111324_WesternNCWater_MSPos\111324_MSPos_Grainger Site 1_47.raw
E:\Orbitrap Raw Data Files\Exploris Raw Files\111324_WesternNCWater_MSPos\111324_MSPos_LRB_21.raw
E:\Orbitrap Raw Data Files\Exploris Raw Files\111324_WesternNCWater_MSPos\111324_MSPos_LRB_51.raw
E:\Orbitrap Raw Data Files\Exploris Raw Files\111324_WesternNCWater_MSPos\111324_MSPos_Marshall Island Standing Water_41.raw
E:\Orbitrap Raw Data Files\Exploris Raw Files\111324_WesternNCWater_MSPos\111324_MSPos_Pool1_56.raw
E:\Orbitrap Raw Data Files\Exploris Raw Files\111324_WesternNCWater_MSPos\111324_MSPos_Pool2_58.raw
E:\Orbitrap Raw Data Files\Exploris Raw Files\111324_WesternNCWater_MSPos\111324_MSPos_Pool3_60.raw
E:\Orbitrap Raw Data Files\Exploris Raw Files\111324_WesternNCWater_MSPos\111324_MSPos_Silver Line_43.raw

----
Processing node 38: Select Spectra
----
1. Spectrum Properties Filter:
- Lower RT Limit: 0
- Upper RT Limit: 0
- First Scan: 0
- Last Scan: 0
- Ignore Specified Scans: (not specified)
- Total Intensity Threshold: 0
- Minimum Peak Count: 1

1.1 Spectrum Properties Filter for DDA Spectra:
- Lowest Charge State: 0
- Highest Charge State: 0
- Min. Precursor Mass: 100 Da
- Max. Precursor Mass: 5000 Da

2. Scan Event Filters:
- Mass Analyzer: (not specified)
- MS Order: Any
- Activation Type: (not specified)
- Acquisition Type: (not specified)
- Min. Collision Energy: 0
- Max. Collision Energy: 1000
- Scan Type: Any
- Polarity Mode: (not specified)
- MS1 Mass Range: (not specified)
- FAIMS CV: (not specified)

3. Peak Filters:
- S/N Threshold (FT-only): 1.5

4. Replacements for Unrecognized Properties:
- Unrecognized Charge Replacements: 1
- Unrecognized Mass Analyzer Replacements: ITMS
- Unrecognized MS Order Replacements: MS2
- Unrecognized Activation Type Replacements: CID
- Unrecognized Polarity Replacements: +
- Unrecognized MS Resolution@200 Replacements: 60000
- Unrecognized MSn Resolution@200 Replacements: 30000

6. General Settings:
- Precursor Selection: Use MS(n - 1) Precursor
- Use Isotope Pattern in Precursor Reevaluation: True
- Provide Profile Spectra: Automatic
- Spectra to Store: All

- Store Chromatograms: False

Processing node 54: Align Retention Times
----
1. General Settings:
- Alignment Model: Adaptive curve
- Alignment Fallback: None
- Maximum Shift [min]: 2
- Shift Reference File: True
- Mass Tolerance: 5 ppm
- Remove Outlier: True

Processing node 53: Detect Compounds
----
1. General Settings:
- Mass Tolerance [ppm]: 5 ppm
- Min. Peak Intensity: 1000000
- Min. # Scans per Peak: 5
- Use Most Intense Isotope Only: True
- Precursor Mass Tolerance: 0.025 Da

2. Trace Detection:
- Max. Number of Gaps to Correct: 2
- Min. Number of Adjacent Non-Zeros: 2
- Trace Mass Update Strategy: Weighted Mean

3. Peak Detection:
- Chromatographic S/N Threshold: 1.5
- Remove Baseline: False
- Gap Ratio Threshold: 0.35
- Max. Peak Width [min]: 1
- Min. Relative Valley Depth: 0.1

4. Isotope Pattern Detection:
- Group Isotopes for: Br; Cl
- RT Tolerance [min]: 0
- Use Peak Quality for Isotope Grouping: True
- Filter out Features with Bad Peaks Only: True
- Zig-Zag Index Threshold: 0.2
- Jaggedness Threshold: 0.4
- Modality Threshold: 0.9
- Remove Potentially False Positive Isotopes: False

5. Compound Assembly:
- Ions:

[2M+ACN+H]+1
[2M+ACN+Na]+1
[2M+FA-H]-1
[2M+H]+1
[2M+K]+1
[2M+Na]+1
[2M+NH4]+1
[2M-H]-1
[2M-H+HAc]-1
[M+2H]+2
[M+3H]+3
[M+ACN+2H]+2
[M+ACN+H]+1
[M+ACN+Na]+1
[M+Cl]-1
[M+DMSO+H]+1
[M+FA-H]-1
[M+H]+1
[M+H+K]+2
[M+H+MeOH]+1
[M+H+Na]+2
[M+H+NH4]+2
[M+H-H2O]+1
[M+H-NH3]+1
[M+K]+1
[M+Na]+1
[M+NH4]+1
[M-2H]-2
[M-2H+K]-1
[M-H]-1
[M-H+HAc]-1
[M-H+TFA]-1
[M-H-H2O]-1
- Base Ions: [M+H]+1; [M+NH4]+1; [M-H]-1
- Remove Singlets: False

6. AcquireX Settings:
- Detect Persistent Background Ions: False

Processing node 25: Group Compounds
----
1. General Settings:
- Mass Tolerance: 5 ppm
- RT Tolerance [min]: 0.1
- Minimum Valley [%]: 10
- Align Peaks: False

- Preferred Ions: [M+H]+1; [M+NH4]+1; [M-2H]-2; [M-H]-1; [M-H-H2O]-1
- Area Integration: Most Common Ion

2. Peak Rating Contributions:
- Area Contribution: 3
- CV Contribution: 10
- FWHM to Base Contribution: 5
- Jaggedness Contribution: 5
- Modality Contribution: 5
- Zig-Zag Index Contribution: 5

3. Peak Rating Filter:
- Peak Rating Threshold: 5
- Number of Files: 2

Processing node 50: Fill Gaps
----
1. General Settings:
- Mass Tolerance: 5 ppm
- S/N Threshold: 1.5
- Use Real Peak Detection: True
- Apply Restrictive Gap Filling: True
- Min. # Scans per Peak: 3

Processing node 55: Apply SERRF QC Correction
----
1. General Settings:
- Min. QC Coverage [%]: 50
- Max. QC Area RSD [%]: 30
- Max. Corrected QC Area RSD [%]: 25
- Max. # Files Between QC Files: 15
- # Batches: 2
- Interpolate Gap-filled QC Areas: True
- Correct Blank Files: False

2. Random Forest Settings:
- # Trees: 200

Processing node 43: Mark Background
----
1. General Settings:
- Max. Sample/Blank: 5
- Max. Blank/Sample: 0
- Hide Background: True

Processing node 46: Calculate Mass Defect
----
1. Mass Defect:
- Fractional Mass: False
- Standard Mass Defect: False
- Relative Mass Defect: False
- Kendrick Mass Defect: True
- Nominal Mass Rounding: Floor

2. Kendrick Formula:
- Formula 1: C2 F4
- Formula 2: C2 F3 O
- Formula 3: C2 H4
- Formula 4: C3 H6
- Formula 5: C8 H8

Processing node 40: Assign Compound Annotations
----
1. General Settings:
- Mass Tolerance: 5 ppm

2. Data Sources:
- Data Source #1: mzCloud Search
- Data Source #2: mzVault Search
- Data Source #3: MassList Search
- Data Source #4: ChemSpider Search
- Data Source #5: Predicted Compositions
- Data Source #6: (not specified)
- Data Source #7: (not specified)

3. Scoring Rules:
- Use mzLogic: True
- Use Spectral Distance: True
- SFit Threshold: 20
- SFit Range: 20

4. Reprocessing:
- Clear Names: False

Processing node 51: Search mzCloud
----
1. General Settings:
- Compound Classes: All

- Precursor Mass Tolerance: 10 ppm
- FT Fragment Mass Tolerance: 10 ppm
- IT Fragment Mass Tolerance: 0.4 Da
- Library: Autoprocessed; Reference
- Post Processing: Recalibrated
- Max. # Results: 10
- Annotate Matching Fragments: True
- Search MSn Tree: False

2. DDA Search:
- Identity Search: Cosine
- Match Activation Type: True
- Match Activation Energy: Match with Tolerance
- Activation Energy Tolerance: 20
- Apply Intensity Threshold: True
- Similarity Search: None
- Match Factor Threshold: 30

3. DIA Search:
- Use DIA Scans for Search: True
- Max. Isolation Width [Da]: 500
- Match Activation Type: False
- Match Activation Energy: Any
- Activation Energy Tolerance: 100
- Apply Intensity Threshold: True
- Match Factor Threshold: 20

Processing node 56: Search mzVault
----
1. Search Settings:
- mzVault Library: 080522 HHEAR POSITIVE and NEGATIVE(d02c6804-04e8-4b2b-ab1e-486e0d84d6da).db|080522 MSMLS POSITIVE and NEGATIVE(b399db47-3f10-473e-9825-49d12c9a2da7).db|080522 NIST 2020 Positive(7b5f4de2-fe8f-41c2-9c6b-19180415e92d).db|062524_EPA ToxCast_NEG_final.db|061724_EPAToxCast_POS_Final.db|051624_CordBlood_FlameRetardants_POS.db|051524_CordBlood_Phenols_POS.db|051424_CordBlood_Phthalates_POS.db|050324_RestekPesticides_POS.db|Pesticides_Exploris_Mzvault_Positive_01102025.db
- Max. # Results: 10
- Match Factor Threshold: 50
- Search Algorithm: HighChem HighRes
- Match Analyzer Type: True
- IT Fragment Mass Tolerance: 0.4 Da
- FT Fragment Mass Tolerance: 10 ppm
- Use Retention Time: False
- Precursor Mass Tolerance: 10 ppm
- Apply Intensity Threshold: True

- Match Ionization Method: True
- Ion Activation Energy Tolerance: 20
- Match Ion Activation Energy: Match with Tolerance
- Match Ion Activation Type: True
- Compound Classes: All
- Remove Precursor Ion: True
- RT Tolerance [min]: 2

Processing node 37: Predict Compositions
----
1. Prediction Settings:
- Mass Tolerance: 5 ppm
- Min. Element Counts: C H
- Max. Element Counts: C90 H190 Br3 Cl8 F18 N10 O18 P3 S5
- Min. RDBE: 0
- Max. RDBE: 40
- Min. H/C: 0.1
- Max. H/C: 3.5
- Max. # Candidates: 10
- Max. # Internal Candidates: 500

2. Pattern Matching:
- Intensity Tolerance [%]: 30
- Intensity Threshold [%]: 0.1
- S/N Threshold: 3
- Min. Spectral Fit [%]: 30
- Min. Pattern Cov. [%]: 80
- Use Dynamic Recalibration: True

3. Fragments Matching:
- Use Fragments Matching: True
- Mass Tolerance: 5 ppm
- S/N Threshold: 3

Processing node 22: Search ChemSpider
----
1. Search Settings:
- Database(s):
    ACToR: Aggregated Computational Toxicology Resource
    DrugBank
    EAWAG Biocatalysis/Biodegradation Database
    EPA DSSTox
    EPA Toxcast
    FDA UNII - NLM
- Search Mode: By Formula or Mass

- Mass Tolerance: 5 ppm
- Max. # of results per compound: 20
- Max. # of Predicted Compositions to be searched per Compound: 3
- Result Order (for Max. # of results per compound): Order By Reference Count (DESC)

2. Predicted Composition Annotation:
- Check All Predicted Compositions: True

Processing node 44: Apply Spectral Distance
----
1. Pattern Matching:
- Mass Tolerance: 5 ppm
- Intensity Tolerance [%]: 30
- Intensity Threshold [%]: 0.1
- S/N Threshold: 3
- Use Dynamic Recalibration: True

Processing node 39: Search Mass Lists
----
1. Search Settings:
- Mass Lists: EFS HRAM Compound Database.masslist|PFAS_NIST.massList|UpdatedPolymerAdditiveList2021v3.massList|EPA_PesticidesList.massList|EPA_ContaminantOfEmergingConcern.massList|Pesticides Mass List.massList
- Use Retention Time: True
- RT Tolerance [min]: 0.5
- Mass Tolerance: 5 ppm

Processing node 14: Merge Features
----
1. Peak Consolidation:
- Mass Tolerance: 5 ppm
- RT Tolerance [min]: 0.1

Processing node 49: Differential Analysis
----
1. General Settings:
- Log10 Transform Values: True
- Group Area Calculation: Median
- Replicate Area Calculation: Median

2. Peak Rating Contributions:
- Update Peak Rating: True
- Area Contribution: 3

- CV Contribution: 10
- FWHM to Base Contribution: 5
- Jaggedness Contribution: 5
- Modality Contribution: 5
- Zig-Zag Index Contribution: 5

REFERENCES

1. Survey, U.S.G. ational Water Information System dataset for French Broad River. Available online: https://waterdata.usgs.gov/ (accessed on July 11, 2025).
2. Martinez, B.; Da Silva, B.F.; Aristizabal-Henao, J.J.; Denslow, N.D.; Osborne, T.Z.; Morrison, E.S.; Bianchi, T.S.; Bowden, J.A. Increased levels of perfluorooctanesulfonic acid (PFOS) during Hurricane Dorian on the east coast of Florida. *Environmental Research* **2022**, *208*, 112635, doi:https://doi.org/10.1016/j.envres.2021.112635.
3. Lin, Y.; Sevillano-Rivera, M.; Jiang, T.; Li, G.; Cotto, I.; Vosloo, S.; Carpenter, C.M.G.; Larese-Casanova, P.; Giese, R.W.; Helbling, D.E.; et al. Impact of Hurricane Maria on Drinking Water Quality in Puerto Rico. *Environmental Science & Technology* **2020**, *54*, 9495-9509, doi:10.1021/acs.est.0c01655.
4. Hedgespeth, M.L.; McCord, J.P.; Phillips, K.A.; Strynar, M.J.; Shea, D.; Nichols, E.G. Suspect-screening analysis of a coastal watershed before and after Hurricane Florence using high-resolution mass spectrometry. *Science of the Total Environment* **2021**, *782*, 146862.
5. Fisher, S.C.; Phillips, P.J.; Brownawell, B.J.; Browne, J.P. Comparison of wastewater-associated contaminants in the bed sediment of Hempstead Bay, New York, before and after Hurricane Sandy. *Marine Pollution Bulletin* **2016**, *107*, 499-508, doi:https://doi.org/10.1016/j.marpolbul.2016.03.044.
